# Prosthetic Joint Infections due to *Candida* Species: A Multicenter International Study

**DOI:** 10.1093/cid/ciae395

**Published:** 2024-08-27

**Authors:** Aurélien Dinh, Martin McNally, Emma D’Anglejan, Christel Mamona Kilu, Julie Lourtet, Rosemary Ho, Matthew Scarborough, Maria Dudareva, Gerald Jesuthasan, Cecile Ronde Oustau, Stéphane Klein, Laura Escolà-Vergé, Dolores Rodriguez Pardo, Pierre Delobel, Jaime Lora-Tamayo, Mikel Mancheño-Losa, Maria Luisa Sorlí Redó, José María Barbero Allende, Cédric Arvieux, Danguole Vaznaisiène, Thomas Bauer, Anne-Laure Roux, Latifa Noussair, Stéphane Corvec, Marta Fernández-Sampedro, Nicolò Rossi, Adrien Lemaignen, Mauro José Costa Salles, Taiana Cunha Ribeiro, Julien Mazet, Milène Sasso, Jean-Philippe Lavigne, Albert Sotto, Etienne Canouï, Éric Senneville, Pauline Thill, Olivier Lortholary, Fanny Lanternier, Laura Morata, Alex Soriano, Gérard Giordano, Camille Fourcade, Bernhard J H Frank, Jochen G Hofstaetter, Clara Duran, Eric Bonnet, for the European Society of Clinical Microbiology and Infectious Diseases Study Group on Implant Associated Infections (ESGIAI), Thomas Bauer, Camille Courboulès, Emma d’Anglejan, Aurélien Dinh, Clara Duran, Christel Mamona Kilu, Latifa Noussair, Anne-Laure Roux, Eric Bonnet, Camille Fourcade, Gérard Giordano, Maria Dudareva, Rosemary Ho, Gerald Jesuthasan, Martin McNally, Matthew Scarborough, Bernhard J H Frank, Jochen G Hofstaetter, Stephane Klein, Cecile Ronde Oustau, Éric Senneville, Pauline Thill, Laura Escolà-Vergé, Dolores Rodriguez Pardo, Laura Morata, Alex Soriano, Etienne Canouï, André Paugam, Gertrude Touanga, Pierre Delobel, Jaime Lora-Tamayo, Mikel Mancheño-Losa, Jean-Philippe Lavigne, Milène Sasso, Julien Mazet, Albert Sotto, Juan Gomez Junyent, Maria Luisa Sorlí Redó, Mauro José Costa Salles, Taiana Cunha Ribeiro, José Maria Barbero Allende, Guillaume Desoubeaux, Adrien Lemaignen, Chloé Porche, Cédric Arvieux, Anne Méheut, Jean-Pierre Gangneux, Carine Couzigou, Julie Lourtet, Benoît Pilmis, Justinas Stucinskas, Danguole Vaznaisiene, Nicolò Rossi, Stéphane Corvec, Vincent Crenn, Florent Morio, Marta Fernández-Sampedro, Fanny Lanternier, Olivier Lortholary

**Affiliations:** Infectious Disease Department, Raymond-Poincaré University Hospital, Paris Saclay University, Assistance Publique-Hôpitaux de Paris, Garches, France; Oxford Bone Infection Unit, Nuffield Orthopaedic Centre, Oxford University Hospitals, Oxford, United Kingdom; Infectious Disease Department, Raymond-Poincaré University Hospital, Paris Saclay University, Assistance Publique-Hôpitaux de Paris, Garches, France; Infectious Disease Department, Raymond-Poincaré University Hospital, Paris Saclay University, Assistance Publique-Hôpitaux de Paris, Garches, France; Clinical Microbiology Laboratory, Saint-Joseph Hospital, Paris; Oxford Bone Infection Unit, Nuffield Orthopaedic Centre, Oxford University Hospitals, Oxford, United Kingdom; Oxford Bone Infection Unit, Nuffield Orthopaedic Centre, Oxford University Hospitals, Oxford, United Kingdom; Oxford Bone Infection Unit, Nuffield Orthopaedic Centre, Oxford University Hospitals, Oxford, United Kingdom; Oxford Bone Infection Unit, Nuffield Orthopaedic Centre, Oxford University Hospitals, Oxford, United Kingdom; Orthopedic Surgery Department, Strasbourg University Hospital, Strasbourg, France; Orthopedic Surgery Department, Strasbourg University Hospital, Strasbourg, France; Infectious Disease Department, Vall d’Hebron University Hospital, Barcelona, Spain; Infectious Disease Department, Vall d’Hebron University Hospital, Barcelona, Spain; Infectious Disease Department, Toulouse University Hospital, Toulouse, France; Internal Medicine Department, Hospital Universitario 12 de Octubre, Madrid; Internal Medicine Department, Hospital Universitario 12 de Octubre, Madrid; Infectious Disease Department, Hospital del Mar, Barcelona; Internal Medicine Department, Hospital Universitario Príncipe de Asturias, Alcalá de Henares, Madrid, Spain; Infectious Disease Department, Rennes University Hospital, Rennes, France; Infectious Disease Department, Lithuanian University of Health Sciences, Kaunas, Lithuania; Orthopedic Surgery Department; Microbiology Department, Raymond-Poincaré University Hospital, Paris Saclay University, Assistance Publique-Hôpitaux de Paris, Garches; Microbiology Department, Raymond-Poincaré University Hospital, Paris Saclay University, Assistance Publique-Hôpitaux de Paris, Garches; Infectious Disease Department, Nantes University Hospital, Nantes, France; Internal Medicine Department, Marques de Valdecilla Hospital, Instituto de Investigación Sanitaria Valdecilla (IDIVAL), Centro De Investigación Biomédica En Red Enfermedades Infecciosas (CIBERINFEC), Santander, Spain; Orthopedic Surgery Department, Sant’Orsola Polyclinic, Bologna, Italy; Infectious Disease Department, Bretonneau University Hospital, Tours, France; Infectious Disease Department, Faculdade de Ciências Médicas Santa Casa de São Paulo, São Paulo, Brazil; Infectious Disease Department, Faculdade de Ciências Médicas Santa Casa de São Paulo, São Paulo, Brazil; Infectious Disease Department, Caremeau University Hospital, Nîmes; Infectious Disease Department, Caremeau University Hospital, Nîmes; Infectious Disease Department, Caremeau University Hospital, Nîmes; Infectious Disease Department, Caremeau University Hospital, Nîmes; Infectious Disease Department, Cochin University Hospital, Assistance Publique-Hôpitaux de Paris, Paris; Infectious Disease Department, Lille University Hospital, Lille; Infectious Disease Department, Lille University Hospital, Lille; Necker-Pasteur Center for Infectious Diseases and Tropical Medicine, Necker-Enfants Malades University Hospital, Assistance Publique-Hôpitaux de Paris, Paris; Mycology Department, Institut Pasteur, Centre National de Référence Mycoses Invasives et Antifongiques, Paris Cité University, Groupe de Recherche Translationnelle en Mycologie, Paris, France; Necker-Pasteur Center for Infectious Diseases and Tropical Medicine, Necker-Enfants Malades University Hospital, Assistance Publique-Hôpitaux de Paris, Paris; Mycology Department, Institut Pasteur, Centre National de Référence Mycoses Invasives et Antifongiques, Paris Cité University, Groupe de Recherche Translationnelle en Mycologie, Paris, France; Infectious Disease Department, Hospital Clínic de Barcelona, Barcelona, Spain; Infectious Disease Department, Hospital Clínic de Barcelona, Barcelona, Spain; Orthopedic surgery department, Joseph Ducuing Hospital, Toulouse, France; Infectious Disease Department, Joseph Ducuing Hospital, Toulouse, France; Michael Ogon Laboratory for Orthopaedic Research, Orthopaedic Hospital Vienna, Speising, Austria; Michael Ogon Laboratory for Orthopaedic Research, Orthopaedic Hospital Vienna, Speising, Austria; Infectious Disease Department, Raymond-Poincaré University Hospital, Paris Saclay University, Assistance Publique-Hôpitaux de Paris, Garches, France; Infectious Disease Department, Joseph Ducuing Hospital, Toulouse, France

**Keywords:** prosthetic joint infection, *Candida* spp, superinfection, mortality, echinocandins

## Abstract

**Background:**

Prosthetic joint infection (PJI) caused by *Candida* spp is a severe complication of arthroplasty. We investigated the outcomes of *Candida* PJI.

**Methods:**

This was a retrospective observational multinational study including patients diagnosed with *Candida*-related PJI between 2010 and 2021. Treatment outcome was assessed at 2-year follow-up.

**Results:**

A total of 269 patients were analyzed. Median age was 73.0 (interquartile range [IQR], 64.0–79.0) years; 46.5% of patients were male and 10.8% were immunosuppressed. Main infection sites were hip (53.0%) and knee (43.1%), and 33.8% patients had fistulas. Surgical procedures included debridement, antibiotics, and implant retention (DAIR) (35.7%), 1-stage exchange (28.3%), and 2-stage exchange (29.0%). *Candida* spp identified were *Candida albicans* (55.8%), *Candida parapsilosis* (29.4%), *Candida glabrata* (7.8%), and *Candida tropicalis* (5.6%). Coinfection with bacteria was found in 51.3% of cases. The primary antifungal agents prescribed were azoles (75.8%) and echinocandins (30.9%), administered for a median of 92.0 (IQR, 54.5–181.3) days. Cure was observed in 156 of 269 (58.0%) cases. Treatment failure was associated with age >70 years (OR, 1.811 [95% confidence interval {CI}: 1.079–3.072]), and the use of DAIR (OR, 1.946 [95% CI: 1.157–3.285]). *Candida parapsilosis* infection was associated with better outcome (OR, 0.546 [95% CI: .305–.958]). Cure rates were significantly different between DAIR versus 1-stage exchange (46.9% vs 67.1%, *P* = .008) and DAIR versus 2-stage exchange (46.9% vs 69.2%, *P* = .003), but there was no difference comparing 1- to 2-stage exchanges (*P* = .777).

**Conclusions:**

*Candida* PJI prognosis seems poor, with high rate of failure, which does not appear to be linked to immunosuppression, use of azoles, or treatment duration.

## Teaser

In this retrospective observational multinational study including patients diagnosed with *Candida*-related prosthetic joint infection (PJI) between 2010 and 2021, 269 patients were analyzed. *Candida* PJI prognosis seems poor and does not appear to be linked to immunosuppression, use of azoles, or treatment duration.

Prosthetic joint infection (PJI) represents 1 of the most severe complications of arthroplasty, with an incidence ranging from 1% to 2% in all arthroplasty procedures [1], imposing a substantial burden on both healthcare systems and individuals [1, 2]. Fungal PJIs are underreported, constituting approximately 1.3% of all PJIs, with *Candida* PJI specifically accounting for >90% of all fungal PJIs [3, 4]. Their incidence has increased in recent decades, attributed to the aging demographic and the rising number of immunosuppressed hosts [5, 6]. Moreover, *Candida* spp have a proclivity for adhering to medical devices and form biofilms, a factor contributing to the persistence and recurrence of these infections [3]. However, the availability of high-quality evidence concerning the optimal management of *Candida* PJI remains largely unknown. Clear-cut guidelines have yet to be established. Consequently, various antifungal and surgical treatments have been documented in the existing literature [3, 7–11]. To date, our understanding of the epidemiology and outcomes associated with *Candida* PJIs remains limited.

Thus, we aimed to describe the epidemiology of *Candida* PJIs, their therapeutic and surgical management, and outcomes, in a large multicenter international cohort study. Moreover, we analyzed the effectiveness of different surgical approaches (prosthesis removal vs retention) and medical treatment strategies (azoles vs antifungals with antibiofilm activity). Finally, we also studied factors associated with failure.

## METHODS

We conducted a large international multicenter retrospective cohort study with the valuable support of the European Study Group for Implant Associated Infections (ESGIAI) of the European Society of Clinical Microbiology and Infectious Diseases (ESCMID). A standardized questionnaire was distributed to all participants and members. This study involved 19 hospitals from 7 countries.

Cases were identified by searching dedicated databases containing consecutive records of PJIs or from the general archives of each participating hospital. Medical records were reviewed by physicians before possible inclusion. All collected data were entered into a central standardized database, which was based on the ESGIAI database. Patients were followed up for a minimum period of 12 months after the completion of their treatment to monitor for any recurrences.

We collected patient-specific information, including patient characteristics, comorbidities, type of surgery, type and duration of systemic antifungal therapy, treatment outcomes, and follow-up duration. All cases underwent a rigorous review process led by C. D. and A. D., with any inconsistencies being addressed by the investigator at each collaborating hospital. Patients were categorized into groups based on the species involved, as well as the surgical and antifungal treatments they received.

### Inclusion Criteria

All cases had PJI confirmed with the 2021 European Bone and Joint Infection Society (EBJIS) definition [12], with microbiological culture of *Candida* spp in 2 or more intraoperative specimens or sterile samples (biopsies, implant culture, or synovial fluid). These patients exhibited signs and symptoms of PJI, such as fever, pain, or other inflammatory indicators, during the period from 2010 to 2021 and received treatment accordingly.

Following previous reports, risk factors for *Candida* PJI included comorbidities associated with any immunocompromised condition [13] (defined as presence of asplenia, neutropenia, agammaglobulinemia, organ transplant, hematological malignancy, known human immunodeficiency virus infection and CD4^+^ count <400 cells/μL, or Child-Pugh class C cirrhosis), prior use of antibiotics, multiple joint surgeries, and previous *Candida* infections.

Echinocandins and amphotericin B were considered effective against biofilms, while azoles lacked this activity [8, 14].

“Cure” was defined as the absence of signs and symptoms of infection after a follow-up period of 2 years. “Treatment failure” was determined by the presence of any of the following: (1) relapse/recurrence, involving the reappearance of clinical signs and symptoms after the initial clinical-surgical treatment, with isolation of the same microorganism or organism not documented; (2) the need for suppressive treatment, assuming that the patient would not be cured with the initial treatment strategy; or (3) death. In cases of relapse, only patients followed up for at least 1 year were included in the analysis.

Categorical variables are presented as absolute frequencies and percentages, while continuous variables are reported as medians and interquartile range (IQR). Differences in percentages between groups were assessed using the χ^2^ test or Fisher exact test for categorical variables. Wilcoxon rank-sum exact tests were used to compare the distributions of quantitative continuous variables. A 2-tailed *P* value <.05 was considered statistically significant.

To identify risk factors associated with failure, univariate analysis by logistic regression was performed using demographic and medical characteristics and clinical, biological, and treatment data. Multivariate analysis by logistic regression was then performed using all variables from the univariate analysis that had a *P* value ≤.05. The final model was obtained using backward stepwise regression with 0.10 thresholds, with Hosmer-Lemeshow statistic calculated to assess the model's goodness of fit and multicollinearity tested. Odds ratios (ORs) were calculated from the univariate and multivariate analysis to quantify the association with failure with 95% confidence intervals (CIs). All statistical analyses were performed using R software, version 4.3.2.

This study received approval from the French Infectious Disease Society Institutional Review Board (IRB00011642). Specific local permissions from the ethics review committee of the promoting center were obtained prior to commencing recruitment. Patient data were anonymized, and all information were handled in accordance with European and local data protection regulations, including General Data Protection Regulation and Commission nationale de l'informatique et des libertés (Reference Method 004) in France.

## RESULTS

Over the 10-year study period, our participating hospitals documented a total of 279 cases of *Candida* PJIs. Ten cases were excluded due to absence of clinical outcome; thus, 269 cases were included in the cohort.

The demographic and clinical profile of the global cohort is presented in Table 1. The median age of the patients was 73.0 (IQR, 64.0–79.0) years. Immunosuppression was observed in 10.8% of cases. The primary infection sites were the hip (53.5%) and knee (43.1%). Patients had undergone a median of 3.0 (IQR, 2.0–5.0) previous surgeries on the same site, and 30.1% of these surgeries occurred within the last month. A previous history of infection at the same site was reported in 75.8% of cases. The most prevalent clinical signs included local inflammatory signs (50.6%), fistulas (33.8%), and dehiscence (30.9%). Overall, 82.4% (75/91) of patients with fistula did receive antibiotic treatments before the diagnosis of fungal PJI.

**Table 1.** Study Patients’ Characteristics

Characteristic	Cure (n = 156)	Failure (n = 113)	Total (n = 269)	*P* Value
Male patients	75 (48.1)	50 (44.2)	125 (46.5)	.534
Age, y, median (IQR)	71.5 (60.8–77.0)	75.0 (68.0–80.0)	73.0 (64.0–79.0)	.003*
BMI, kg/m^2^, median (IQR)	28.4 (24.9–33.8)	30.4 (26.2–35.3)	29.4 (25.2–34.7)	.266
Charlson score, median (IQR)	3.0 (2.0–5.0)	4.0 (2.0–5.0)	4.0 (2.0–5.0)	.053
Immunosuppression	19 (12.2)	10 (8.8)	29 (10.8)	.385
Immunosuppressive treatments	14 (9)	10 (8.8)	24 (8.9)	.989
Diabetes	38 (24.4)	34 (30.1)	72 (26.8)	.295
Localization of prosthesis				
Hip	80 (51.3)	64 (56.6)	144 (53.5)	.385
Knee	69 (44.2)	47 (41.6)	116 (43.1)	.666
Shoulder	2 (1.3)	2 (1.8)	4 (1.5)	.744
Tibia (knee hemiprosthesis)	2 (1.3)	0 (0)	2 (0.7)	.227
Femur (hip hemiprosthesis)	2 (1.3)	0 (0)	2 (0.7)	.227
Ankle	1 (0.6)	0 (0)	1 (0.4)	1.000
No. of previous surgeries, median (IQR)	3.0 (2.0–5.0)	3.0 (2.0–5.0)	3.0 (2.0–5.0)	.297
No. of previous surgeries due to infection, median (IQR)	1.0 (0.0–2.0)	1.0 (1.0–3.0)	1.0 (0.0–2.0)	.310
Time between previous surgery and index infection, d, median (IQR)	79.0 (32.5–543.8)	43.0 (21.0–234.5)	58.0 (24.0–383.3)	.008*
Previous surgery <1 mo	36 (23.1)	45 (39.8)	81 (30.1)	.004*
Previous surgery <3 mo	77 (49.4)	70 (61.9)	147 (54.6)	.046
History of previous infection				
Previous infection	116 (74.4)	88 (77.9)	204 (75.8)	.506
Previous infection due to *Candida* spp	9 (5.8)	4 (3.5)	13 (4.8)	.376
Microbiology analysis of previous infections				
Mono-bacterial infection	52 (33.3)	41 (36.3)	93 (34.6)	.737
*Staphylococcus* sp	77 (49.4)	56 (49.6)	133 (49.4)	.748
*Staphylococcus aureus*	33 (21.2)	22 (19.5)	55 (20.4)	.611
Coagulase-negative staphylococci	54 (34.6)	38 (33.6)	92 (34.2)	.674
*Streptococcus* sp	4 (2.6)	6 (5.3)	10 (3.7)	.328
*Enterococcus* sp	16 (10.3)	18 (15.9)	34 (12.6)	.190
*Acinetobacter* sp	2 (1.3)	1 (0.9)	3 (1.1)	1.000
*Pseudomonas aeruginosa*	13 (8.3)	14 (12.4)	27 (10)	.308
Enterobacterales	40 (25.6)	23 (20.4)	63 (23.4)	.212
Corynebacteria	5 (3.2)	7 (6.2)	12 (4.5)	.232
Anaerobes	9 (5.8)	10 (8.8)	19 (7.1)	.317
Previous antibiotic therapy	111 (71.2)	84 (74.3)	195 (72.5)	.755
No. of lines of antibiotics, median (IQR)	1.0 (1.0–2.0)	1.0 (1.0–2.0)	1.0 (1.0–2.0)	.249
Clinical signs				
Fever	25 (16)	21 (18.6)	46 (17.1)	.487
Inflammatory signs	74 (47.4)	62 (54.9)	136 (50.6)	.109
Purulent discharge	4 (2.6)	3 (2.7)	7 (2.6)	1.000
Dehiscence	43 (27.6)	40 (35.4)	83 (30.9)	.118
Fistula	52 (33.3)	39 (34.5)	91 (33.8)	.781
Hematoma	24 (15.4)	17 (15)	41 (15.2)	.977
Biological analysis, median (IQR)				
Leukocyte count, g/L	7.5 (6.1–8.9)	7.6 (6.3–9.5)	7.6 (6.1–9.1)	.406
Neutrophil count, g/L	5.2 (3.6–6.6)	5.0 (3.9–6.4)	5.1 (3.7–6.6)	.807
C-reactive protein level, mg/L	34.9 (14.4–66.3)	36.9 (14.0–89.3)	35.0 (14.0–73.0)	.802
ESR, mm/h	48.5 (37.3–68.5)	66.0 (34.0–90.0)	64.0 (35.0–85.0)	.540
Albumin level, g/L	31.7 (24.0–36.0)	29.5 (20.3–35.0)	31.0 (22.8–36.0)	.230
Radiographic evidence of infection				
X-ray	61 (39.1)	43 (38.1)	104 (38.7)	.843
CT scan	15 (9.6)	14 (12.4)	29 (10.8)	.529
Scintigraphy	2 (1.3)	3 (2.7)	5 (1.9)	.387
Loosening	21 (13.5)	13 (11.5)	34 (12.6)	.644
Abscess	13 (8.3)	13 (11.5)	26 (9.7)	.283
TTE/TOE	7 (4.5)	9 (8)	16 (5.9)	.356
Signs of endocarditis	0 (0)	1 (0.9)	1 (0.4)	.396
Type of surgery				
DAIR	45 (28.8)	51 (45.1)	96 (35.7)	.004*
1-stage exchange	51 (32.7)	25 (22.1)	76 (28.3)	.071
2-stage exchange	54 (34.6)	24 (21.2)	78 (29)	.022*
Girdlestone resection	3 (1.9)	4 (3.5)	7 (2.6)	.454
Prosthesis removal	2 (1.3)	2 (1.8)	4 (1.5)	1.000
Amputation	1 (0.6)	1 (0.9)	2 (0.7)	1.000
Microbiology analysis of index infection				
Positive blood culture	19 (12.2)	13 (11.5)	32 (11.9)	.895
Pluri-microbial	75 (48.1)	51 (45.1)	126 (46.8)	.598
Only due to *Candida* spp	74 (47.4)	57 (50.4)	131 (48.7)	.626
*Candida albicans*	78 (50)	72 (63.7)	150 (55.8)	.025*
*Candida glabrata*	11 (7.1)	10 (8.8)	21 (7.8)	.587
*Candida parapsilosis*	54 (34.6)	25 (22.1)	79 (29.4)	.026*
*Candida tropicalis*	10 (6.4)	5 (4.4)	15 (5.6)	.484
*Candida dubliniensis*	1 (0.6)	2 (1.8)	3 (1.1)	.574
*Candida metapsilosis*	3 (1.9)	1 (0.9)	4 (1.5)	.641
*Candida orthopsilosis*	2 (1.3)	0 (0)	2 (0.7)	.511
*Candida krusei*	1 (0.6)	1 (0.9)	2 (0.7)	1.000
*Candida kefyr*	1 (0.6)	0 (0)	1 (0.4)	1.000
*Candida lusitaniae*	1 (0.6)	0 (0)	1 (0.4)	1.000
Coinfection with bacteria				
*Staphylococcus* sp	40 (25.6)	33 (29.2)	73 (27.1)	.541
*Staphylococcus aureus*	13 (8.3)	12 (10.6)	25 (9.3)	.538
Coagulase-negative staphylococci	32 (20.5)	22 (19.5)	54 (20.1)	.802
*Enterococcus* sp	13 (8.3)	7 (6.2)	20 (7.4)	.495
*Streptococcus* sp	1 (0.6)	1 (0.9)	2 (0.7)	1.000
Enterobacterales	27 (17.3)	10 (8.8)	37 (13.8)	.043*
*Escherichia coli*	8 (5.1)	3 (2.7)	11 (4.1)	.365
*Klebsiella pneumoniae*	7 (4.5)	3 (2.7)	10 (3.7)	.525
*Enterobacter* sp	8 (5.1)	3 (2.7)	11 (4.1)	.365
*Proteus* sp	2 (1.3)	1 (0.9)	3 (1.1)	1.000
*Pseudomonas aeruginosa*	6 (3.8)	4 (3.5)	10 (3.7)	1.000
*Acinetobacter baumannii*	0 (0)	1 (0.9)	1 (0.4)	.422
*Stenotrophomonas maltophilia*	2 (1.3)	2 (1.8)	4 (1.5)	1.000
Corynebacteria	6 (3.8)	7 (6.2)	13 (4.8)	.384
Anaerobes	5 (3.2)	4 (3.5)	9 (3.3)	1.000
*Neisseria macacae*	1 (0.6)	0 (0)	1 (0.4)	1.000
Antifungal susceptibility testing				
Resistance to fluconazole	10 (6.4)	11 (9.7)	21 (7.8)	.327
Resistance to voriconazole	6 (3.8)	5 (4.4)	11 (4.1)	1.000
Resistance to posaconazole	4 (2.6)	1 (0.9)	5 (1.9)	.639
Resistance to AmB	2 (1.3)	2 (1.8)	4 (1.5)	1.000
Resistance to echinocandins	21 (13.5)	8 (7.1)	29 (10.8)	.169
Resistance to 5-fluorocytosine	3 (1.9)	2 (1.8)	5 (1.9)	1.000
Resistance to itraconazole	0 (0)	1 (0.9)	1 (0.4)	.300
Antifungal treatments				
Azoles	116 (74.4)	88 (77.9)	204 (75.8)	.884
Echinocandins	46 (29.5)	37 (32.7)	83 (30.9)	.777
Azoles & echinocandins	9 (5.8)	7 (6.2)	16 (5.9)	.972
AmB	11 (7.1)	8 (7.1)	19 (7.1)	.910
5-flucytosine	1 (0.6)	0 (0)	1 (0.4)	1.000
Echinocandins & 5-flucytosine	2 (1.3)	4 (3.5)	6 (2.2)	.407
Echinocandins & AmB	1 (0.6)	2 (1.8)	3 (1.1)	.580
Azoles & 5-flucytosine	5 (3.2)	1 (0.9)	6 (2.2)	.238
Azoles & AmB	0 (0)	1 (0.9)	1 (0.4)	.433
No. of lines, median (IQR)	1.0 (1.0–2.0)	1.0 (1.0–2.0)	1.0 (1.0–2.0)	.799
Antifungal treatment duration, d, median (IQR)	92.0 (59.8–177.0)	92.5 (45.8–187.8)	92.0 (54.5–181.3)	.968
Antifungal duration <6 wk	18 (15.5)	18 (22.5)	36 (18.4)	.215
Antifungal duration 6–12 wk	29 (25.0)	15 (18.8)	44 (22.4)	.303
Antifungal duration >12 wk	69 (59.5)	47 (58.8)	116 (59.2)	.918
Percentage of total treatment time, median (IQR)				
Azoles	100.0 (83.1–100.0)	100.0 (86.0–100.0)	100.0 (85.0–100.0)	.905
Echinocandins	21.7 (8.1–100.0)	34.2 (12.8–100.0)	24.2 (9.4–100.0)	.429
Azoles & echinocandins	12.8 (8.0–14.0)	20.4 (12.4–34.3)	13.4 (9.6–26.7)	.181
AmB	14.5 (13.2–26.9)	19.2 (9.3–44.7)	14.5 (10.5–28.1)	.607
Echinocandins & 5-flucytosine	19.3 (18.7–19.9)	75.7 (41.1–100.0)	35.9 (18.7–87.8)	.481
Echinocandins & AmB	15.0 (15.0–15.0)	2.1 (2.1–2.1)	8.5 (5.3–11.7)	>.999
Azoles & 5-flucytosine	61.1 (56.4–74.2)	21.6 (21.6–21.6)	58.7 (30.3–70.9)	.667
Azoles & AmB	…	12.4 (12.4–12.4)	12.4 (12.4–12.4)	
Antifungal spacer	8 (5.1)	4 (3.5)	12 (4.5)	.533
Spacer voriconazole	4 (2.6)	1 (0.9)	5 (1.9)	.576
Spacer AmB	4 (2.6)	2 (1.8)	6 (2.2)	1.000
Spacer fluconazole	0 (0)	1 (0.9)	1 (0.4)	.333

Data are presented as No. (%) unless stated otherwise.

Abbreviations: AmB, amphotericin B; BMI, body mass index; CT, computed tomography; DAIR, debridement, antibiotics, and implant retention; ESR, erythrocyte sedimentation rate; IQR, interquartile range; TOE, transesophageal echocardiogram; TTE, transthoracic echocardiogram.

^*^Statistically significant.

In terms of surgical strategy, debridement, antibiotics, and implant retention (DAIR) were performed in 35.7% of cases, while 1- or 2-stage exchange procedures were executed in 28.3% and 29.0%, respectively.

In the microbiological analysis, the most frequently identified *Candida* spp were *Candida albicans* (55.8%) and *Candida parapsilosis* (29.4%). Coinfection with bacteria was observed in 51.3% of cases, with *Staphylococcus aureus* (20.4%), coagulase-negative staphylococci (34.2%), Enterobacterales (23.4%), and *Enterococcus* spp (12.6%) being the predominant bacterial species involved. Notably, 72.5% of patients had previously received antibiotic treatment in the last 3 months.

Regarding surgical and medical therapy, the primary antifungal agents employed were azoles (75.8%) and echinocandins (30.9%). The median duration of antifungal treatment was 92.0 (IQR, 54.5–181.3) days. A combination of antifungal therapies was utilized in 9.1% of cases.

Study outcomes are described in Table 2. Overall, cure after a follow-up period of 1 year was observed in 58.0% of cases.

**Table 2.** Description of Study Outcomes

Outcome	No. (%) (N = 269)
Cure	156 (58.0)
Recurrence other germs	52 (19.3)
Failure	113 (42.0)
Suppressive treatment	18 (6.7)
Recurrence	52 (19.3)
Recurrence to *Candida* spp	21 (7.8)
Recurrence not documented	18 (6.7)
Recurrence to *Candida* spp and other bacteria	14 (5.2)
Death due to infectious cause	34 (12.6)
Death due to other cause	9 (3.3)
Prosthesis removal	74 (27.5)

In our univariate analysis, we identified significant differences between patients who achieved a cure and those who experienced treatment failure based on age, the use of DAIR, and *C. parapsilosis* infection (complete results in Supplementary Table 1). In the multivariate analysis (Table 3), factors associated with treatment failure were age >70 years (OR, 1.811 [95% CI: 1.079–3.072], *P* = .026), DAIR (OR, 1.946 [95% CI: 1.157–3.285], *P* = .012) as the surgical approach, while the presence of *C. parapsilosis* rather than other *Candida* spp was associated with a favorable outcome (OR, 0.546 [95% CI: .305–.958], *P* = .037).

**Table 3.** Univariable and Multivariable Analysis of Factors Associated With Failure

Factor	Univariable Analysis			Multivariable Analysis		
	OR	(95% CI)	*P* Value	OR	(95% CI)	*P* Value
Age >70 y	1.881	(1.140–3.138)	.014	1.811	(1.079–3.072)	.026
DAIR	2.113	(1.271–3.534)	.004	1.946	(1.157–3.285)	.012
Presence of *Candida parapsilosis*	0.537	(.305–.925)	.027	0.546	(.305–.958)	.037

Abbreviations: CI, confidence interval; DAIR, debridement, antibiotics, and implant retention; OR, odds ratio.

We performed a subgroup analysis of the population without bacterial coinfection (n = 131 [46.9%]), presented in Supplementary Tables 2 and 3. Median age was 74.0 (IQR, 66.0–79.0) years, with a male to female ratio of 0.48; 9.9% were immunosuppressed. The main antifungal therapies were azoles (88.3%) and echinocandins (30.6%), and main surgical strategies were DAIR in 35 cases (27.6%) and 1- or 2-stage exchange in 81 cases (63.8%). The multivariate analysis performed to identify factors associated with failure found that DAIR was also associated with failure, while infection due to *C. parapsilosis* seemed protective (Supplementary Table 4).

To explore the impact of surgery on the whole cohort, we performed sensitivity analyses comparing patients with DAIR versus 1- or 2-stage exchange (Supplementary Tables 5–8). There was a significant difference regarding outcome comparing DAIR versus 1-stage exchange and DAIR versus 2-stage exchange (*P* = .008 and *P* = .003, respectively), but there was no difference comparing 1- to 2-stage exchanges.

DAIR was more frequently performed in cases with previous surgery in the last month (*P* < .001) and coinfection with bacteria (*P* = .036). However, recent surgery was an independent factor associated with failure in the univariate analysis (*P* = .004), contrary to coinfection with bacteria (*P* = .626) (Supplementary Table 1).

Exchange surgery was more frequently performed in case of radiographic loosening (*P* < .001), prosthetic loosening being always a contraindication to DAIR, which requires a stable implant, and was associated with better outcome due to a lower infection recurrence rate (*P* < .001).

Furthermore, the sensitivity analysis comparing outcome according to treatment duration (6–12 weeks vs >12 weeks) did not find any significant difference regarding outcome and population characteristics (Supplementary Tables 9–11).

Finally, the susceptibility to antifungal agents according to species showed that *C. albicans* versus non-*C. albicans* and *C. parapsilosis* versus non-*C. parapsilosis* were not significantly different (Supplementary Table 12).

## DISCUSSION

In this international multicenter study, we present the largest cohort of PJI due to *Candida* spp to the best of our knowledge, using the 2021 EBJIS definition of PJI and microbiological identification and a 2-year follow-up to assess outcome [12].

*Candida* PJI is a rare condition that poses a therapeutic challenge. In our series, the infection predominantly affected older patients with various comorbidities. However, less than 11% of patients had evidence of immunosuppression. The main clinical presentation was indolent, occurring among patients with numerous previous local surgeries and/or infections, which is consistent with previous small series [15–17]. The only risk factors associated with treatment failure were age and retention of the prosthesis. Moreover, involvement of *C. albicans* had a higher failure rate than *C. parapsilosis* (48.0% vs 31.6%, respectively).

*Candida albicans* was the most common microorganism isolated, followed by *C. parapsilosis*, which is in accordance with the global epidemiology of *Candida* PJI found in the literature [8, 18]. Two multicenter studies in Spain [9, 19] and 1 study in the United States [7] found similar results: *C. albicans* was isolated in 55%–65% of cases, and *C. parapsilosis* in 13%–33% cases. Nonetheless, epidemiology of the species of *Candida* may still vary among regions.

Usual risks factors for *Candida* infection, including immunosuppression, systemic disease, and/or long-term antibiotic use, may play an essential role in the development of invasive *Candida* infections. We found few immunosuppressed patients in our study, but most patients had previously been exposed to antibiotics and had several surgeries on the infected site. These risk factors have been previously described in the literature and appear to be the most important risk factors for PJI due to *Candida* [4, 15–17].

*Candida* PJIs are usually chronic infections characterized by pain, swelling, and sinus tracts, with implant loosening observed on radiography or computed tomography in nearly 50% of cases [9]. Fever is rarely found, as in our results. Because symptoms are mild, the diagnosis can often be delayed. Also, the presence of *Candida* spp in samples could be considered as a contaminant, since there is still no standard definition focused on fungal PJI, which is a potential limitation of our study, particularly when bacterial coinfection is present.

This limitation was addressed by using strict inclusion criteria, requiring 2 positive cultures of the same fungus, from intraoperative specimens, direct biopsies, or synovial fluid, which fulfills the EBJIS definition of PJI as confirmed infection [12].

The treatment used was successful in 58% of patients, although removal of the implant was the only strategy significantly associated with success. These results are concordant with literature data [9]. Few reports have described successful treatment with retention of the prosthesis, but these infections were treated in the acute stage or had a follow-up shorter than a year [15, 20, 21]. A literature review of hip PJI due to *Candida* collected 79 cases through 35 articles [22]. It revealed a preference for 2-stage revision (44.9%) and fluconazole as medical therapy (73.5%), and suggested better clinical outcome with 1- or 2-stage revision than with resection arthroplasty or debridement, though with a low level of evidence.

In chronic infections, irrigation and debridement alone with prosthesis retention failed to control the infection [7, 16]. Most authors prefer to remove all the infected material, in accordance with the Infectious Diseases Society of America (IDSA) guidelines [23]. The reported cure rates were very variable (14% to almost 100%) when performing 1- or 2-stage exchange arthroplasty combined with antifungal agents [7, 15, 16, 24–27]. In our study, prosthesis exchange was also associated with better outcome than DAIR.

Interestingly, in our study, antifungals with antibiofilm activity and long treatment duration with antifungal therapy were not associated with better outcome. We suggest that antibiofilm activity, if the prosthesis is removed, has no impact. Indeed, better antibiofilm activity has been demonstrated in vitro, but the clinical effect remains under debate.

Moreover, median treatment duration was shorter than usually recommended. Indeed, treatment duration is not well supported by studies, and patient tolerance to antifungal treatment toxicity could often lead to discontinuation.

According to previous reports, 6–12 months of treatment seemed necessary, particularly when azoles were used [28–32]. Recent IDSA guidelines recommend prosthesis removal and at least 3 months of antifungals, but the evidence for this recommendation is scarce [23]. In a small series, shorter antifungal courses (eg, 6 weeks) were highly successful when using a 2-stage exchange procedure [25]. In our study, the median duration of antifungal treatment was 3 months, similar to that reported in other series [7, 24, 25]. Further studies should determine whether treatment duration <3 months is successful when antibiofilm agents are used, particularly in combination with implant removal. Nonetheless, our study did not advocate for prolonged systemic use of antibiofilm fungal therapy, which could lead to substantial economic savings while avoiding adverse events and fungal resistance.

Cement spacers impregnated with antifungal drugs are sometimes recommended. In our study, this was uncommon, with only 6.5% of cases having a spacer (19% of all 2-stage revisions). Their use is still controversial. It could be of interest due to the high concentration in the site without systemic absorption, which may prevent possible adverse events.

The success rate of *Candida* PJI was low, and treatment with prosthesis removal led to a better outcome. Indeed, DAIR with the absence of prosthesis exchange was a major factor associated with failure, probably due to the ability of *Candida* spp to set up biofilms. Therefore, in a chronic prosthesis infection, exchange surgery seems mandatory, though *Candida* PJI mostly occurred in patients with comorbidities and numerous previous surgeries, which could limit the possible prosthesis exchange, especially outside of referral centers [33].

The other risk factor for failure was age, which is a well-known risk factor that could limit surgery but is not specific to *Candida* PJI [34]. A recent systematic review included 71 patients with hip PJI and 126 with knee PJI [11]. In this work, risk factors for recurrence were knee prosthesis, Charlson score >3, *C. albicans* PJI, and C-reactive protein level ≥6 mg/L. Compared to DAIR, 2-stage exchange was a protective factor for PJI recurrence in the knee. No risk factors were found in patients who had hip PJI. The DAIR procedure and the *C. albicans* etiology are in line with our results.

Additionally, *C. albicans*, which is the main species involved in *Candida* infection and especially PJI, was associated with a lower cure rate. This could be due to the inverse collinearity with *C. parapsilosis*. Indeed, infection due to *C. parapsilosis* seemed less severe than due to *C. albicans*, as demonstrated for bloodstream infections [32, 35]. This difference could partly be due to both species having different inflammatory pathways [36]. Moreover, a small retrospective study comparing PJI due to *C. albicans* versus non-*C. albicans* found that the infection-free survival rate decreased in patients with *C. albicans* PJI, which underlined the possible role of the causative pathogen [37].

The main limitations of our study are due to its retrospective design and the possible heterogeneity of the management due to the large time period of our study and the numerous different countries that participated. We included patients treated over an extended period, which may have resulted in variability in several factors such as the surgeons, surgical techniques, and antifungal regimens available. In addition, there could be recall bias. Moreover, the analysis may have been underpowered to detect some significant differences. Also, in our study, we did not collect any megaprosthesis fungal PJIs, which are rare but have devastating outcome and often lead to amputation, as recently reported [38]. Last, we included patients with PJI solely due to *Candida* but also patients with superinfection/coinfection due to bacteria, which could be a confounding factor.

Nonetheless, it is a multicenter study, carried out in centers with multidisciplinary teams and meticulous recording of data on these patients, that does reflect the scope of therapy being used for *Candida* PJI currently. Although the sample size is moderate, it is the largest series of its kind published in recent years. Only a clinical trial could correctly address the therapeutic questions. However, due to the rare incidence of *Candida* PJI, performing a randomized controlled trial with correct sample size remains challenging.

## CONCLUSIONS

*Candida* PJI occurred mainly among patients with numerous previous surgeries, and clinical presentation was indolent. The main species involved were *C. albicans* and *C. parapsilosis*. The prognosis was poor and did not appear to be linked to immunosuppression, use of azoles, or treatment duration. Factors associated with treatment failure included age and absence of prosthesis removal. Infection due to *Candida parapsilosis* seemed to have a better prognosis.

## Supplementary Data

Supplementary materials are available at *Clinical Infectious Diseases* online. Consisting of data provided by the authors to benefit the reader, the posted materials are not copyedited and are the sole responsibility of the authors, so questions or comments should be addressed to the corresponding author.

## Supplementary Material

ciae395_Supplementary_Data

## Notes

***Author contributions.*** A. D. had full access to all of the data in the study and takes responsibility for the integrity of the data and the accuracy of the data analysis. Concept and design: A. D., C. D., and E. B. Acquisition, analysis, or interpretation of data: All authors. Drafting of the manuscript: A. D., M. M., C. D., and E. B. Critical revision of the manuscript for important intellectual content: All authors. Statistical analysis: A. D. and C. D.. Supervision: A. D., M. M., J. L.-T., C. D., and E. B.

***Acknowledgments.*** The authors acknowledge as co-authors all of the following ESGIAI members: Raymond-Poincaré University Hospital, Paris Saclay University, Assistance Publique-Hôpitaux de Paris (AP-HP), Garches, France: Thomas Bauer, Camille Courboulès, Emma d’Anglejan, Aurélien Dinh, Clara Duran, Christel Mamona Kilu, Latifa Noussair, Anne-Laure Roux; Joseph Ducuing Hospital, Toulouse, France: Eric Bonnet, Camille Fourcade, Gérard Giordano; Nuffield Orthopaedic Centre, Oxford University Hospitals, Oxford, UK: Maria Dudareva, Rosemary Ho, Gerald Jesuthasan, Martin McNally, Matthew Scarborough; Michael Ogon Laboratory for Orthopaedic Research, Orthopaedic Hospital Vienna, Speising, Austria: Bernhard J. H. Frank, Jochen G. Hofstaetter; Strasbourg University Hospital, Strasbourg, France: Stephane Klein, Cecile Ronde Oustau; Lille University Hospital, Lille, France: Éric Senneville, Pauline Thill; Vall d’Hebron University Hospital, Barcelona, Spain: Laura Escolà-Vergé, Dolores Rodriguez Pardo; Hospital Clínic de Barcelona, Spain: Hospital Clinic de Barcelona, Barcelona, Spain: Laura Morata, Alex Soriano; Cochin University Hospital, AP-HP, Paris, France: Etienne Canouï, André Paugam, Gertrude Touanga; Toulouse University Hospital, Toulouse, France: Pierre Delobel; Hospital Universitario 12 de Octubre, Madrid, Spain: Jaime Lora-Tamayo, Mikel Mancheño-Losa; Caremeau University Hospital, Nîmes, France: Jean-Philippe Lavigne, Milène Sasso, Julien Mazet, Albert Sotto; Hospital del Mar, Barcelona, Spain: Juan Gomez Junyent, Maria Luisa Sorlí Redó; Faculdade de Ciências Médicas Santa Casa de São Paulo, Brasil: Mauro José Costa Salles, Taiana Cunha Ribeiro; Príncipe de Asturias University Hospital, Alcalá de Henares, Madrid, Spain: José Maria Barbero Allende; Bretonneau University Hospital, Tours, France: Guillaume Desoubeaux, Adrien Lemaignen, Chloé Porche; Rennes University Hospital, Rennes, France: Cédric Arvieux, Anne Méheut, Jean-Pierre Gangneux; Saint-Joseph Hospital, Paris, France: Carine Couzigou, Julie Lourtet, Benoît Pilmis; Lithuanian University of Health Sciences, Kaunas, Lituania: Justinas Stucinskas, Danguole Vaznaisiene; Sant’Orsola Polyclinic, Bologna, Italy: Nicolò Rossi; Nantes University Hospital, Nantes, France: Stéphane Corvec, Vincent Crenn, Florent Morio; Marques de Valdecilla Hospital—IDIVAL, CIBERINFEC, Santander, Spain: Marta Fernández-Sampedro; Necker-Pasteur Center for Infectious Diseases and Tropical Medicine, Necker-Enfants Malades University Hospital, AP-HP, Paris, France; Institut Pasteur, Centre National de Référence Mycoses Invasives et Antifongiques, Paris Cité University, Groupe de Recherche Translationnelle en Mycologie, Mycology Department, Paris, France: Fanny Lanternier, Olivier Lortholary.

***Data availability.*** The datasets used and/or analyzed during the current study are available from the corresponding author on reasonable request.
