# Correction to: Prosthetic Joint Infections due to *Candida* Species: A Multicenter International Study

**DOI:** 10.1093/cid/ciae492

**Published:** 2024-10-10

An error appeared in the corrected proof publication of this article (Dinh et al. “Prosthetic Joint Infections due to *Candida* Species: A Multicenter International Study.” *Clin Infect Dis*. https://doi.org/10.1093/cid/ciae395). Two of the authors’ names were misspelled. Jochen G. Hofstätter should be **Jochen G Hofstaetter**, and Bernhard J. H. Franck should be **Bernhard JH Frank**.

