# Supplementary material for: Prosthetic Joint Infections due to Candida Species: A Multicenter International Study
Source: Clin Infect Dis. 2024 Aug 27;80(2):347–55. doi: 10.1093/cid/ciae395 (PMC11848259; doi:10.1093/cid/ciae395)
Supplement: ciae395_Supplementary_Data [file ciae395_supplementary_data.docx]

**Supplementary appendix**

[I. Univariable analysis of factors associated with failure among the study population 2](#_Toc168345319)

[Table S1. Univariable analysis of factors associated with failure among the study population (n=269) 2](#_Toc168345320)

[II. Analyses on *Candida* PJI without bacterial co-infection 5](#_Toc168345321)

[Table S2. Patients’ characteristics among population with PJI due to *Candida* spp. with *versus* without bacterial co-infection 5](#_Toc168345322)

[Table S3. Patients’ characteristics among population with PJI due to *Candida* spp. without bacterial co-infection (n=131) 8](#_Toc168345323)

[Table S4. Univariable and multivariable analyses of factors associated with failure 11](#_Toc168345324)

[III. Analyses on *Candida* PJI depending on type of surgery 12](#_Toc168345325)

[Table S5. DAIR *versus* Exchange surgery 12](#_Toc168345326)

[Table S6. DAIR *versus* One-stage exchange 16](#_Toc168345327)

[Table S7. DAIR *versus* Two-stage exchange 20](#_Toc168345328)

[Table S8. One-stage exchange *versus* Two-stage exchange 24](#_Toc168345329)

[IV. Analysis on *Candida* PJI depending on antifungal treatment duration 27](#_Toc168345330)

[Table S9. Patient's characteristics according to antifungal duration (< 6 weeks *versus* 6-12 weeks) 27](#_Toc168345331)

[Table S10. Patients' characteristics according to antifungal duration (< 6 weeks *versus* > 12 weeks) 31](#_Toc168345332)

[Table S11. Patients' characteristics according to antifungal duration (6-12 weeks *versus* > 12 weeks) 35](#_Toc168345333)

[V. Analysis on antifungal sensibility according to *Candida* species 39](#_Toc168345334)

[Table S12. Antifungal sensibility analysis according to *Candida* species 39](#_Toc168345335)

## Univariable analysis of factors associated with failure among the study population

Table S1. Univariable analysis of factors associated with failure among the study population (n=269)

| **Variable** | **OR**^1^ | **95% CI**^1^ | **p-value** |
| --- | --- | --- | --- |
| Male patients | 0.857 | 0.526, 1.393 | 0.534 |
| Age (years) | 1.029 | 1.008, 1.052 | 0.009* |
| Age > 70 yo | 1.881 | 1.140, 3.138 | 0.014* |
| Body Mass Index | 0.997 | 0.973, 1.004 | 0.549 |
| Charlson score | 1.125 | 1.009, 1.259 | 0.036* |
| Immunosuppression | 0.700 | 0.301, 1.540 | 0.387 |
| Immunosuppressive treatments | 0.994 | 0.413, 2.315 | 0.989 |
| Diabetes | 1.336 | 0.775, 2.303 | 0.296 |
| **Localization of prosthesis** |  |  |  |
| Hip | 1.241 | 0.763, 2.024 | 0.385 |
| Knee | 0.898 | 0.549, 1.464 | 0.666 |
| Other | 0.384 | 0.056, 1.623 | 0.238 |
| Number of previous surgeries | 1.043 | 0.943, 1.154 | 0.407 |
| Number of previous surgeries due to infection | 1.058 | 0.926, 1.211 | 0.404 |
| Time between previous surgery and index infection | 1.000 | 0.999, 1.000 | 0.064 |
| Previous Surgery < 1 month | 2.213 | 1.295, 3.813 | 0.004* |
| Previous Surgery < 3 months | 1.692 | 1.011, 2.858 | 0.047 |
| Previous infection | 1.214 | 0.689, 2.169 | 0.506 |
| Previous infection due to *Candida* spp. | 0.582 | 0.153, 1.853 | 0.381 |
| **Microbiology analysis of previous infections** |  |  |  |
| Mono-bacterial infection | 1.104 | 0.620, 1.968 | 0.737 |
| *Staphylococcus* sp. | 0.902 | 0.481, 1.700 | 0.748 |
| *Staphylococcus aureus* | 0.847 | 0.444, 1.598 | 0.611 |
| Coagulase-negative staphylococci | 0.884 | 0.495, 1.573 | 0.674 |
| *Streptococcus* sp | 2.160 | 0.602, 8.632 | 0.242 |
| *Enterococcus* sp. | 1.643 | 0.779, 3.495 | 0.192 |
| *Acinetobacter* sp. | 0.663 | 0.030, 7.033 | 0.739 |
| *Pseudomonas aeruginosa* | 1.527 | 0.672, 3.494 | 0.310 |
| Enterobacterales | 0.674 | 0.359, 1.247 | 0.213 |
| Corynebacteria | 2.022 | 0.628, 7.004 | 0.240 |
| Anaerobes | 1.609 | 0.626, 4.193 | 0.320 |
| Previous antibiotic therapy | 1.261 | 0.301, 6.287 | 0.755 |
| Previous ATB duration | 1.005 | 1.000, 1.012 | 0.101 |
| **Clinical signs** |  |  |  |
| Fever | 1.255 | 0.656, 2.384 | 0.488 |
| Inflammatory signs | 1.504 | 0.914, 2.488 | 0.109 |
| Purulent discharge | 0.888 | 0.171, 4.148 | 0.879 |
| Dehiscence | 1.522 | 0.897, 2.586 | 0.119 |
| Fistula | 1.076 | 0.640, 1.803 | 0.781 |
| Hematoma | 0.990 | 0.496, 1.942 | 0.977 |
| **Biological analysis** |  |  |  |
| Leucocyte count (G/L) | 1.026 | 0.966, 1.095 | 0.406 |
| Neutrophil count (G/L) | 1.013 | 0.916, 1.120 | 0.799 |
| C-Reactive Protein level (mg/L) | 1.002 | 0.998, 1.006 | 0.361 |
| ESR (mm/h) | 1.008 | 0.987, 1.030 | 0.462 |
| Albumin level (g/L) | 0.973 | 0.936, 1.009 | 0.149 |
| **Radiographic evidence of infection** |  |  |  |
| Loosening | 0.835 | 0.381, 1.777 | 0.644 |
| Abscess | 1.576 | 0.679, 3.663 | 0.286 |
| Signs of endocarditis | 3,299,473 | 0.000, NA | 0.986 |
| **Type of surgery** |  |  |  |
| DAIR | 2.113 | 1.271, 3.534 | 0.004* |
| Exchange | 0.383 | 0.230, 0.631 | <0.001* |
| One-stage exchange | 0.600 | 0.340, 1.040 | 0.072 |
| Two-stage exchange | 0.522 | 0.294, 0.906 | 0.023* |
| Other type of surgery | 3.000 | 1.034, 9.872 | 0.051 |
| **Microbiology analysis of index infection** |  |  |  |
| Positive blood culture | 0.951 | 0.439, 2.004 | 0.895 |
| Pluri-microbial | 0.877 | 0.539, 1.426 | 0.598 |
| Only due to *Candida* spp. | 1.128 | 0.695, 1.833 | 0.626 |
| *C. albicans* | 1.756 | 1.073, 2.896 | 0.026* |
| *C. parapsilosis* | 0.537 | 0.305, 0.925 | 0.027* |
| Other *Candida* spp. | 0.885 | 0.462, 1.663 | 0.707 |
| **Co-infection with bacteria** | 0.887 | 0.546, 1.440 | 0.626 |
| *Staphylococcus* sp. | 1.187 | 0.683, 2.055 | 0.541 |
| *S. aureus* | 1.296 | 0.559, 2.982 | 0.539 |
| Coagulase-negative staphylococci | 0.925 | 0.497, 1.698 | 0.802 |
| *Enterococcus* sp | 0.718 | 0.261, 1.820 | 0.497 |
| *Streptococcus* sp | 1.371 | 0.054, 34.94 | 0.824 |
| Enterobacterales | 0.455 | 0.201, 0.960 | 0.046 |
| *E. coli* | 0.499 | 0.107, 1.772 | 0.313 |
| *K. pneumoniae* | 0.574 | 0.122, 2.119 | 0.430 |
| *Enterobacter* sp. | 0.499 | 0.107, 1.772 | 0.313 |
| *Proteus* sp. | 0.681 | 0.031, 7.199 | 0.755 |
| *Pseudomonas aeruginosa* | 0.908 | 0.227, 3.262 | 0.884 |
| *Acinetobacter baumannii* | 2,925,106 | 0.000, NA | 0.987 |
| *Stenotrophomonas maltophilia* | 1.375 | 0.163, 11.61 | 0.752 |
| Corynebacteria | 1.638 | 0.529, 5.230 | 0.388 |
| Anaerobes | 1.098 | 0.266, 4.248 | 0.891 |
| **Antifungal susceptibility testing** |  |  |  |
| Resistance to Fluconazole | 1.563 | 0.632, 3.905 | 0.330 |
| Resistance to Voriconazole | 1.111 | 0.311, 3.809 | 0.866 |
| Resistance to Posaconazole | 0.348 | 0.017, 2.545 | 0.359 |
| Resistance to Amphotericin B | 1.389 | 0.164, 11.75 | 0.745 |
| Resistance to Echinocandins | 0.547 | 0.217, 1.262 | 0.174 |
| Resistance to 5-fluorocytosine | 0.867 | 0.110, 5.467 | 0.879 |
| **Antifungal treatments** |  |  |  |
| Antibiofilm > 1 week | 0.995 | 0.570, 1.727 | 0.985 |
| Azoles | 0.948 | 0.466, 1.958 | 0.884 |
| Echinocandins | 1.080 | 0.631, 1.846 | 0.777 |
| Azoles & Echinocandins | 1.018 | 0.353, 2.828 | 0.972 |
| Amphotericin B | 0.947 | 0.354, 2.430 | 0.910 |
| Echinocandins & 5-flucytosine | 2.680 | 0.513, 19.61 | 0.260 |
| Echinocandins & Amphotericin B | 2.647 | 0.250, 57.42 | 0.429 |
| Azoles & 5-flucytosine | 0.254 | 0.013, 1.609 | 0.215 |
| Duration < 6 weeks | 1.581 | 0.762, 3.285 | 0.217 |
| Duration between 6 and 12 weeks | 0.692 | 0.337, 1.379 | 0.304 |
| Duration > 12 weeks | 0.970 | 0.544, 1.736 | 0.918 |
| Antifungal-impregnated cement spacer | 0.679 | 0.178, 2.213 | 0.536 |

^1^OR = Odds Ratio, CI = Confidence Interval

* Statistically significant

## Analyses on *Candida* PJI without bacterial co-infection

Table S2. Patients’ characteristics among population with PJI due to *Candida* spp. with *versus* without bacterial co-infection

|  | With Bacterial Co-infection  N = 138^1^ | Without Bacterial Co-infection  N = 131^1^ | P-value^2^ |
| --- | --- | --- | --- |
| Age (year) | 72.0 (63.3 - 79.0) | 74.0 (66.0 - 79.0) | 0.324 |
| Age > 70 yo | 75 (54.3%) | 85 (64.9%) | 0.078 |
| Body Mass Index | 29.4 (25.2 - 35.2) | 29.6 (25.2 - 33.3) | 0.681 |
| Charlson score | 3.0 (2.0 - 5.0) | 4.0 (2.3 - 5.0) | 0.451 |
| Male patients | 62 (44.9%) | 63 (48.1%) | 0.603 |
| Immunosuppression | 16 (11.6%) | 13 (9.9%) | 0.659 |
| Immunosuppressive treatments | 15 (11.2%) | 9 (7.4%) | 0.295 |
| Diabetes | 33 (23.9%) | 39 (29.8%) | 0.278 |
| **Localization of prosthesis** |  |  |  |
| Hip | 82 (59.4%) | 62 (47.3%) | 0.047* |
| Knee | 52 (37.7%) | 64 (48.9%) | 0.064 |
| Other | 4 (2.9%) | 5 (3.8%) | 0.744 |
| Number of previous surgeries | 3.0 (2.0 - 5.0) | 3.0 (2.0 - 5.0) | 0.036* |
| Number of previous surgeries due to infection | 1.0 (1.0 - 2.0) | 1.0 (0.0 - 2.0) | 0.084 |
| Time between previous surgery and index infection | 42.0 (24.0 - 173.0) | 84.0 (28.0 - 560.0) | 0.037* |
| Previous Surgery < 1 month | 47 (36.4%) | 34 (28.1%) | 0.159 |
| Previous Surgery < 3 months | 84 (65.1%) | 63 (52.1%) | 0.036* |
| Previous infection | 109 (79.0%) | 95 (72.5%) | 0.216 |
| Previous infection due to *Candida* spp. | 4 (3.6%) | 9 (9.5%) | 0.081 |
| **Microbiology analysis of previous infections** |  |  |  |
| Mono-bacterial infection | 50 (49.5%) | 43 (48.9%) | 0.930 |
| Previous antibiotic therapy | 101 (92.7%) | 94 (100.0%) | 0.008* |
| Number of lines of ATB | 1.0 (1.0 - 2.0) | 1.0 (1.0 - 2.0) | 0.342 |
| Previous ATB duration | 42.0 (24.0 - 74.0) | 52.0 (41.5 - 82.5) | 0.067 |
| **Clinical signs** |  |  |  |
| Fever | 26 (20.0%) | 20 (15.6%) | 0.359 |
| Inflammatory signs | 66 (50.4%) | 70 (54.7%) | 0.488 |
| Purulent discharge | 7 (7.8%) | 0 (0.0%) | 0.014* |
| Dehiscence | 47 (36.4%) | 36 (28.1%) | 0.154 |
| Fistula | 54 (41.2%) | 37 (28.9%) | 0.038* |
| Hematoma | 24 (19.0%) | 17 (13.5%) | 0.232 |
| **Biological analysis** |  |  |  |
| Leucocyte count (G/L) | 7.6 (6.3 - 9.2) | 7.6 (5.9 - 8.7) | 0.545 |
| Neutrophil count (G/L) | 5.1 (3.8 - 6.3) | 5.0 (3.6 - 7.0) | 0.886 |
| C-Reactive Protein level (mg/L) | 37.8 (16.3 - 74.7) | 27.3 (11.8 - 69.6) | 0.136 |
| ESR (mm/h) | 59.0 (36.5 - 86.5) | 65.0 (36.5 - 83.8) | 0.829 |
| Albumin level (g/L) | 30.0 (22.5 - 34.5) | 31.0 (23.0 - 37.1) | 0.425 |
| **Radiographic evidence of infection** |  |  |  |
| X-ray | 46 (39.7%) | 58 (49.6%) | 0.128 |
| CT scan | 15 (30.6%) | 14 (32.6%) | 0.841 |
| Scintigraphy | 1 (2.4%) | 4 (11.1%) | 0.179 |
| Loosening | 14 (14.9%) | 20 (22.7%) | 0.175 |
| Abscess | 10 (10.8%) | 16 (18.8%) | 0.128 |
| TTE/TOE | 10 (11.6%) | 6 (8.0%) | 0.443 |
| Signs of endocarditis | 0 (0.0%) | 1 (1.7%) | 0.448 |
| **Type of surgery** |  |  |  |
| DAIR | 61 (44.2%) | 35 (27.6%) | 0.005* |
| Exchange surgery | 73 (52.9%) | 81 (63.8%) | 0.073 |
| One-stage exchange | 36 (26.1%) | 40 (31.5%) | 0.331 |
| Two-stage exchange | 37 (26.8%) | 41 (32.3%) | 0.329 |
| Other type of surgery | 4 (2.9%) | 11 (8.7%) | 0.043* |
| **Microbiology analysis of index infection** |  |  |  |
| Positive blood culture | 19 (14.6%) | 13 (10.1%) | 0.267 |
| Pluri-microbial | 121 (88.3%) | 5 (3.8%) | <0.001* |
| *C. albicans* | 78 (56.5%) | 72 (55.0%) | 0.797 |
| *C. parapsilosis* | 38 (27.5%) | 41 (31.3%) | 0.498 |
| Other *Candida* spp. | 27 (19.6%) | 21 (16.0%) | 0.449 |
| **Antifungal susceptibility testing** |  |  |  |
| Resistance to Fluconazole | 8 (7.1%) | 13 (10.7%) | 0.327 |
| Resistance to Voriconazole | 4 (4.1%) | 7 (6.4%) | 0.454 |
| Resistance to Posaconazole | 2 (8.0%) | 3 (8.6%) | >0.999 |
| Resistance to Amphotericin B | 2 (1.9%) | 2 (1.8%) | >0.999 |
| Resistance to Echinocandins | 12 (12.2%) | 17 (16.3%) | 0.406 |
| **Antifungal treatments** |  |  |  |
| Number of lines | 1.0 (1.0 - 2.0) | 1.0 (1.0 - 2.0) | 0.008 |
| Antibiofilm > 1 week | 42 (32.6%) | 32 (28.8%) | 0.533 |
| Azoles | 106 (82.2%) | 98 (88.3%) | 0.186 |
| Echinocandins | 49 (38.0%) | 34 (30.6%) | 0.232 |
| Azoles & Echinocandins | 5 (3.9%) | 11 (9.9%) | 0.062 |
| Amphotericin B | 8 (6.2%) | 11 (9.9%) | 0.289 |
| Antifungal treatment duration | 87.0 (44.3 - 170.8) | 114.5 (62.0 - 186.8) | 0.009* |
| Antifungal duration ≤ 6 weeks | 26 (24.5%) | 10 (11.1%) | 0.016* |
| Antifungal duration between 6 and 12 weeks | 23 (21.7%) | 21 (23.3%) | 0.785 |
| Antifungal duration ≥ 12 weeks | 57 (53.8%) | 59 (65.6%) | 0.094 |
| Antifungal-impregnated cement spacer | 4 (2.9%) | 8 (6.1%) | 0.203 |
| **Outcome** |  |  |  |
| Cure | 82 (59.4%) | 74 (56.5%) | 0.626 |
| Recurrence other germs | 28 (20.3%) | 24 (18.3%) | 0.683 |
| Failure | 56 (40.6%) | 57 (43.5%) | 0.626 |
| Suppressive treatment | 8 (5.8%) | 10 (7.6%) | 0.547 |
| Recurrence | 24 (17.4%) | 28 (21.4%) | 0.408 |
| Recurrence to *Candida* spp. | 11 (8.0%) | 10 (7.6%) | 0.918 |
| Recurrence not documented | 6 (4.3%) | 12 (9.2%) | 0.114 |
| Recurrence to *Candida* spp. and other bacteria | 7 (5.1%) | 7 (5.3%) | 0.920 |
| Death due to infectious cause | 18 (13.0%) | 16 (12.2%) | 0.838 |
| Death due to other cause | 6 (4.3%) | 3 (2.3%) | 0.502 |
| Follow-up period | 487.5 (236.0 - 1,074.3) | 637.5 (293.3 - 1,171.5) | 0.431 |

^1^ Median (IQR1 - IQR3); n (%)

^2^ Pearson's Chi-squared test; Fisher's exact test; Wilcoxon rank sum exact test

*Statistically significant

Table S3. Patients’ characteristics among population with PJI due to *Candida* spp. without bacterial co-infection (n=131)

|  | Cure  N = 74^1^ | Failure  N = 57^1^ | P-value^2^ |
| --- | --- | --- | --- |
| Age (year) | 73.0 (61.0 - 78.0) | 74.0 (69.0 - 80.0) | 0.156 |
| Age > 70 yo | 44 (59.5%) | 41 (71.9%) | 0.138 |
| Body Mass Index | 28.3 (24.9 - 32.1) | 31.5 (26.4 - 35.4) | 0.133 |
| Charlson score | 3.0 (2.0 - 5.0) | 4.0 (3.0 - 5.5) | 0.166 |
| Male patients | 33 (44.6%) | 30 (52.6%) | 0.361 |
| Immunosuppression | 10 (13.5%) | 3 (5.3%) | 0.117 |
| Immunosuppressive treatments | 7 (10.3%) | 2 (3.7%) | 0.296 |
| Diabetes | 18 (24.3%) | 21 (36.8%) | 0.120 |
| **Localization of prosthesis** |  |  |  |
| Hip | 33 (44.6%) | 29 (50.9%) | 0.475 |
| Knee | 38 (51.4%) | 26 (45.6%) | 0.515 |
| Other | 3 (4.1%) | 2 (3.5%) | >0.999 |
| Number of previous surgeries | 3.0 (1.3 - 5.0) | 3.0 (2.0 - 4.0) | 0.930 |
| Number of previous surgeries due to infection | 1.0 (0.0 - 2.0) | 1.0 (1.0 - 2.0) | 0.196 |
| Time between previous surgery and index infection | 180.0 (37.0 - 596.0) | 52.0 (21.0 - 254.3) | 0.012 |
| Previous Surgery < 1 month | 14 (20.3%) | 20 (38.5%) | 0.028 |
| Previous Surgery < 3 months | 29 (42.0%) | 34 (65.4%) | 0.011 |
| Previous infection | 51 (68.9%) | 44 (77.2%) | 0.293 |
| Previous infection due to *Candida* spp. | 7 (13.7%) | 2 (4.5%) | 0.170 |
| **Microbiology analysis of previous infections** |  |  |  |
| Mono-bacterial infection | 23 (47.9%) | 20 (50.0%) | 0.846 |
| *Staphylococcus* sp. | 35 (72.9%) | 23 (57.5%) | 0.129 |
| *Staphylococcus aureus* | 12 (25.0%) | 7 (17.5%) | 0.395 |
| Coagulase-negative staphylococci | 27 (56.3%) | 16 (40.0%) | 0.129 |
| *Streptococcus* sp | 1 (1.4%) | 4 (7.5%) | 0.163 |
| *Enterococcus* sp. | 6 (12.5%) | 9 (22.5%) | 0.214 |
| *Acinetobacter* sp. | 2 (4.2%) | 1 (2.5%) | >0.999 |
| *Pseudomonas aeruginosa* | 3 (6.3%) | 9 (22.5%) | 0.027 |
| Enterobacterales | 18 (37.5%) | 13 (32.5%) | 0.625 |
| Corynebacteria | 1 (1.4%) | 2 (3.8%) | 0.575 |
| Anaerobes | 4 (5.6%) | 5 (9.4%) | 0.495 |
| Previous antibiotic therapy | 50 (100.0%) | 44 (100.0%) |  |
| Number of lines of ATB | 2.0 (1.0 - 2.0) | 1.0 (1.0 - 2.0) | 0.301 |
| Previous ATB duration | 56.0 (40.5 - 81.8) | 47.5 (42.0 - 81.8) | 0.718 |
| **Clinical signs** |  |  |  |
| Fever | 7 (9.6%) | 13 (23.6%) | 0.030 |
| Inflammatory signs | 33 (45.2%) | 37 (67.3%) | 0.013 |
| Purulent discharge | 0 (0.0%) | 0 (0.0%) |  |
| Dehiscence | 16 (22.2%) | 20 (35.7%) | 0.092 |
| Fistula | 18 (25.0%) | 19 (33.9%) | 0.269 |
| Hematoma | 9 (12.7%) | 8 (14.5%) | 0.761 |
| **Biological analysis** |  |  |  |
| Leucocyte count (G/L) | 7.3 (5.7 - 8.4) | 7.9 (6.4 - 9.6) | 0.098 |
| Neutrophil count (G/L) | 4.7 (3.6 - 6.9) | 5.2 (3.9 - 7.1) | 0.707 |
| C-Reactive Protein level (mg/L) | 25.0 (11.8 - 55.1) | 38.0 (11.9 - 85.9) | 0.347 |
| ESR (mm/h) | 47.0 (35.0 - 68.0) | 66.0 (42.0 - 85.0) | 0.570 |
| Albumin level (g/L) | 32.0 (25.5 - 37.5) | 30.0 (20.3 - 36.6) | 0.350 |
| **Radiographic evidence of infection** |  |  |  |
| X-ray | 32 (48.5%) | 26 (51.0%) | 0.789 |
| CT scan | 6 (26.1%) | 8 (40.0%) | 0.331 |
| Scintigraphy | 2 (10.0%) | 2 (12.5%) | >0.999 |
| Loosening | 10 (20.8%) | 10 (25.0%) | 0.642 |
| Abscess | 7 (14.9%) | 9 (23.7%) | 0.303 |
| TTE/TOE | 2 (5.3%) | 4 (10.8%) | 0.430 |
| Signs of endocarditis | 0 (0.0%) | 1 (3.8%) | 0.433 |
| **Type of surgery** |  |  |  |
| DAIR | 14 (19.2%) | 21 (38.9%) | 0.014 |
| Exchange surgery | 57 (78.1%) | 24 (44.4%) | <0.001 |
| One-stage exchange | 29 (39.7%) | 11 (20.4%) | 0.020 |
| Two-stage exchange | 28 (38.4%) | 13 (24.1%) | 0.089 |
| Other type of surgery | 2 (2.7%) | 9 (16.7%) | 0.009 |
| **Microbiology analysis of index infection** |  |  |  |
| Positive blood culture | 4 (5.6%) | 9 (15.8%) | 0.055 |
| Pluri-microbial | 3 (4.1%) | 2 (3.5%) | >0.999 |
| Only due to *Candida* spp. | 74 (100.0%) | 57 (100.0%) |  |
| *C. albicans* | 34 (45.9%) | 38 (66.7%) | 0.018 |
| *C. parapsilosis* | 30 (40.5%) | 11 (19.3%) | 0.009 |
| Other *Candida* spp. | 12 (16.2%) | 9 (15.8%) | 0.947 |
| **Antifungal susceptibility testing** |  |  |  |
| Resistance to Fluconazole | 6 (8.7%) | 7 (13.5%) | 0.402 |
| Resistance to Voriconazole | 4 (6.6%) | 3 (6.3%) | >0.999 |
| Resistance to Posaconazole | 3 (13.0%) | 0 (0.0%) | 0.536 |
| Resistance to Amphotericin B | 1 (1.5%) | 1 (2.1%) | >0.999 |
| Resistance to Echinocandins | 13 (20.3%) | 4 (10.0%) | 0.166 |
| **Antifungal treatments** |  |  |  |
| Number of lines | 2.0 (1.0 - 2.0) | 1.0 (1.0 - 2.0) | 0.586 |
| Antibiofilm > 1 week | 17 (28.8%) | 15 (28.8%) | 0.997 |
| Azoles | 52 (88.1%) | 46 (88.5%) | 0.958 |
| Echinocandins | 19 (32.2%) | 15 (28.8%) | 0.702 |
| Azoles & Echinocandins | 6 (10.2%) | 5 (9.6%) | 0.922 |
| Amphotericin B | 6 (10.2%) | 5 (9.6%) | 0.922 |
| **Percentage of total treatment time** |  |  |  |
| Azoles | 95.7 (74.9 - 100.0) | 97.9 (82.6 - 100.0) | 0.617 |
| Echinocandins | 17.5 (5.2 - 72.1) | 18.6 (9.9 - 78.3) | 0.584 |
| Azoles & Echinocandins | 9.6 (5.6 - 12.9) | 31.9 (23.1 - 38.1) | 0.067 |
| Amphotericin B | 26.9 (14.5 - 29.2) | 14.5 (6.4 - 41.6) | 0.905 |
| Antifungal treatment duration | 93.5 (62.0 - 169.5) | 139.0 (65.5 - 208.0) | 0.100 |
| Antifungal duration ≤ 6 weeks | 6 (12.0%) | 4 (10.0%) | >0.999 |
| Antifungal duration between 6 and 12 weeks | 14 (28.0%) | 7 (17.5%) | 0.242 |
| Antifungal duration ≥ 12 weeks | 30 (60.0%) | 29 (72.5%) | 0.215 |
| Antifungal-impregnated cement spacer | 6 (8.1%) | 2 (3.5%) | 0.465 |
| **Outcome** |  |  |  |
| Cure |  |  |  |
| Recurrence other germs | 24 (32.4%) |  |  |
| Failure |  |  |  |
| Suppressive treatment |  | 10 (17.5%) |  |
| Recurrence |  | 28 (49.1%) |  |
| Recurrence to *Candida* spp. |  | 10 (17.5%) |  |
| Recurrence not documented |  | 12 (21.1%) |  |
| Recurrence to *Candida* spp. and other bacteria |  | 7 (12.3%) |  |
| Death due to infectious cause |  | 16 (28.1%) |  |
| Death due to other cause |  | 3 (5.3%) |  |
| Follow-up period | 740.0 (491.0 - 1,244.0) | 380.0 (78.0 - 808.0) | <0.001 |

^1^ Median (IQR1 - IQR3); n (%)

^2^ Pearson's Chi-squared test; Fisher's exact test; Wilcoxon rank sum exact test

*Statistically significant

Table S4. Univariable and multivariable analyses of factors associated with failure

|  | **Univariable analysis** | | | | **Multivariable analysis** | | |
| --- | --- | --- | --- | --- | --- | --- | --- |
| **Variable** | **N** | **OR**^1^ | **95% CI**^1^ | **p-value** | **OR**^1^ | **95% CI**^1^ | **p-value** |
| Age > 70 yo | 131 | 1.747 | 0.840, 3.723 | 0.140 | 1.546 | 0.706, 3.450 | 0.280 |
| DAIR | 127 | 2.682 | 1.217, 6.071 | 0.016 | 2.391 | 1.053, 5.562 | 0.039 |
| *C. parapsilosis* | 131 | 0.351 | 0.152, 0.767 | 0.011 | 0.370 | 0.156, 0.832 | 0.019 |

^1^OR = Odds Ratio, CI = Confidence Interval

DAIR: debridement, antibiotics, and implant retention

## Analyses on *Candida* PJI depending on type of surgery

Table S5. DAIR *versus* Exchange surgery

|  | Exchange Surgery  N = 154^1^ | DAIR  N = 96^1^ | Overall  N = 250^1^ | P-value^2^ |
| --- | --- | --- | --- | --- |
| Age (years) | 71.0 (61.0 - 79.0) | 74.0 (67.8 - 78.0) | 73.0 (63.3 - 78.8) | 0.210 |
| Age > 70 yo | 82 (53.2%) | 63 (65.6%) | 145 (58.0%) | 0.054 |
| Body Mass Index | 28.9 (25.2 - 33.9) | 30.0 (26.1 - 34.9) | 29.4 (25.3 - 34.4) | 0.587 |
| Charlson Score | 3.5 (2.0 - 5.0) | 4.0 (2.0 - 5.0) | 4.0 (2.0 - 5.0) | 0.688 |
| Male patients | 74 (48.1%) | 42 (43.8%) | 116 (46.4%) | 0.507 |
| Immunosuppression | 17 (11.0%) | 11 (11.5%) | 28 (11.2%) | 0.919 |
| Immunosuppressive treatments | 11 (7.5%) | 12 (13.3%) | 23 (9.7%) | 0.140 |
| Diabetes | 34 (22.1%) | 32 (33.3%) | 66 (26.4%) | 0.050 |
| **Localization of prosthesis** |  |  |  |  |
| Hip | 77 (50.0%) | 54 (56.3%) | 131 (52.4%) | 0.336 |
| Knee | 72 (46.8%) | 39 (40.6%) | 111 (44.4%) | 0.343 |
| Other | 5 (3.2%) | 3 (3.1%) | 8 (3.2%) | >0.999 |
| Number of previous surgeries | 3.0 (2.0 - 5.0) | 4.0 (2.0 - 5.5) | 3.0 (2.0 - 5.0) | 0.007* |
| Number of previous surgeries due to infection | 1.0 (0.0 - 2.0) | 2.0 (1.0 - 3.0) | 1.0 (0.0 - 2.0) | <0.001* |
| Time between previous surgery and index infection | 130.0 (38.0 - 587.0) | 33.0 (20.0 - 77.5) | 59.0 (25.8 - 385.3) | <0.001* |
| Previous Surgery < 1 month | 29 (20.6%) | 46 (48.4%) | 75 (31.8%) | <0.001* |
| Previous Surgery < 3 months | 66 (46.8%) | 73 (76.8%) | 139 (58.9%) | <0.001* |
| Previous infection | 110 (71.4%) | 80 (83.3%) | 190 (76.0%) | 0.032* |
| Previous infection due to *Candida* spp. | 7 (6.3%) | 3 (3.7%) | 10 (5.2%) | 0.523 |
| **Microbiology analysis of previous infections** |  |  |  |  |
| Mono-bacterial infection | 56 (56.0%) | 30 (40.0%) | 86 (49.1%) | 0.036* |
| *Staphylococcus* sp. | 69 (69.0%) | 55 (73.3%) | 124 (70.9%) | 0.532 |
| *Staphylococcus aureus* | 31 (31.0%) | 22 (29.3%) | 53 (30.3%) | 0.812 |
| Coagulase-negative staphylococci | 49 (49.0%) | 36 (48.0%) | 85 (48.6%) | 0.896 |
| *Streptococcus* sp | 8 (5.6%) | 1 (1.1%) | 9 (3.8%) | 0.159 |
| *Enterococcus* sp. | 14 (14.0%) | 15 (20.0%) | 29 (16.6%) | 0.291 |
| *Pseudomonas aeruginosa* | 8 (8.0%) | 16 (21.3%) | 24 (13.7%) | 0.011 |
| *Enterobacterales* | 25 (25.0%) | 32 (42.7%) | 57 (32.6%) | 0.014 |
| Corynebacteria | 7 (4.9%) | 4 (4.4%) | 11 (4.7%) | >0.999 |
| Anaerobes | 8 (5.6%) | 9 (9.9%) | 17 (7.2%) | 0.211 |
| Previous antibiotic therapy | 103 (95.4%) | 78 (97.5%) | 181 (96.3%) | 0.701 |
| Number of lines of ATB | 1.0 (1.0 - 2.0) | 1.0 (1.0 - 2.0) | 1.0 (1.0 - 2.0) | 0.505 |
| Previous ATB duration | 47.0 (29.0 - 87.0) | 48.0 (28.8 - 74.0) | 47.0 (28.5 - 84.0) | 0.643 |
| **Clinical signs** |  |  |  |  |
| Fever | 26 (17.6%) | 17 (18.7%) | 43 (18.0%) | 0.828 |
| Inflammatory signs | 72 (49.3%) | 50 (53.2%) | 122 (50.8%) | 0.558 |
| Purulent discharge | 5 (5.2%) | 2 (3.1%) | 7 (4.3%) | 0.704 |
| Dehiscence | 36 (24.8%) | 42 (44.7%) | 78 (32.6%) | 0.001* |
| Fistula | 44 (29.9%) | 38 (40.9%) | 82 (34.2%) | 0.082 |
| Hematoma | 12 (8.5%) | 25 (27.2%) | 37 (15.8%) | <0.001* |
| **Biological analysis** |  |  |  |  |
| Leucocyte count (G/L) | 7.6 (6.3 - 9.2) | 7.5 (5.9 - 8.8) | 7.6 (6.0 - 9.2) | 0.519 |
| Neutrophil count (G/L) | 5.0 (3.7 - 6.6) | 5.1 (3.5 - 6.3) | 5.0 (3.6 - 6.5) | 0.707 |
| C-Reactive Protein level (mg/L) | 29.0 (13.6 - 66.0) | 43.0 (14.7 - 80.6) | 35.0 (14.2 - 72.5) | 0.179 |
| ESR (mm/h) | 59.0 (39.5 - 92.5) | 68.0 (32.5 - 83.5) | 64.0 (36.5 - 87.5) | 0.903 |
| Albumin level (g/L) | 30.0 (23.0 - 36.0) | 31.0 (22.0 - 35.0) | 31.0 (23.0 - 35.0) | 0.868 |
| **Radiographic evidence of infection** |  |  |  |  |
| X-ray | 57 (44.5%) | 39 (43.3%) | 96 (44.0%) | 0.861 |
| CT scan | 17 (28.8%) | 10 (34.5%) | 27 (30.7%) | 0.588 |
| Scintigraphy | 5 (10.0%) | 0 (0.0%) | 5 (6.8%) | 0.167 |
| Loosening | 30 (28.8%) | 2 (2.9%) | 32 (18.6%) | <0.001* |
| Abscess | 16 (15.2%) | 8 (12.3%) | 24 (14.1%) | 0.594 |
| **Microbiology analysis of index infection** |  |  |  |  |
| Positive blood culture | 15 (10.3%) | 13 (13.7%) | 28 (11.7%) | 0.431 |
| Pluri-microbial | 65 (42.5%) | 55 (57.3%) | 120 (48.2%) | 0.023* |
| Only due to *Candida* spp. | 81 (52.6%) | 35 (36.5%) | 116 (46.4%) | 0.013* |
| *C. albicans* | 77 (50.0%) | 58 (60.4%) | 135 (54.0%) | 0.108 |
| *C. parapsilosis* | 52 (33.8%) | 24 (25.0%) | 76 (30.4%) | 0.143 |
| Other *Candida* spp. | 26 (16.9%) | 19 (19.8%) | 45 (18.0%) | 0.560 |
| **Co-infection with bacteria** | 73 (47.4%) | 61 (63.5%) | 134 (53.6%) | 0.013* |
| *Staphylococcus* sp. | 37 (25.5%) | 34 (37.8%) | 71 (30.2%) | 0.047* |
| *S. aureus* | 16 (11.0%) | 7 (7.8%) | 23 (9.8%) | 0.414 |
| Coagulase-negative staphylococci | 24 (16.6%) | 29 (32.2%) | 53 (22.6%) | 0.005* |
| *Enterococcus* sp | 8 (5.5%) | 10 (11.1%) | 18 (7.7%) | 0.117 |
| *Enterobacterales* | 16 (11.0%) | 20 (22.2%) | 36 (15.3%) | 0.021* |
| *E. coli* | 4 (2.8%) | 6 (6.7%) | 10 (4.3%) | 0.188 |
| *K. pneumoniae* | 6 (4.1%) | 4 (4.4%) | 10 (4.3%) | >0.999 |
| *Enterobacter* sp. | 2 (1.4%) | 9 (10.0%) | 11 (4.7%) | 0.003* |
| *Pseudomonas aeruginosa* | 5 (3.4%) | 4 (4.4%) | 9 (3.8%) | 0.735 |
| Corynebacteria | 10 (6.9%) | 3 (3.3%) | 13 (5.5%) | 0.380 |
| **Antifungal susceptibility testing** |  |  |  |  |
| Resistance to Fluconazole | 10 (7.7%) | 10 (11.5%) | 20 (9.2%) | 0.343 |
| Resistance to Voriconazole | 3 (2.7%) | 7 (8.9%) | 10 (5.2%) | 0.095 |
| Resistance to Posaconazole | 4 (10.3%) | 1 (5.9%) | 5 (8.9%) | >0.999 |
| Resistance to Echinocandins | 17 (15.3%) | 11 (14.5%) | 28 (15.0%) | 0.874 |
| Resistance to 5-fluorocytosine | 2 (3.4%) | 3 (9.1%) | 5 (5.5%) | 0.349 |
| **Antifungal treatments** |  |  |  |  |
| Number of lines | 1.0 (1.0 - 2.0) | 1.0 (1.0 - 2.0) | 1.0 (1.0 - 2.0) | 0.410 |
| Antibiofilm > 1 week | 35 (25.4%) | 37 (42.5%) | 72 (32.0%) | 0.007 |
| Azoles | 118 (85.5%) | 73 (83.9%) | 191 (84.9%) | 0.744 |
| Echinocandins | 44 (31.9%) | 37 (42.5%) | 81 (36.0%) | 0.105 |
| Azoles & Echinocandins | 9 (6.5%) | 4 (4.6%) | 13 (5.8%) | 0.547 |
| Amphotericin B | 11 (8.0%) | 8 (9.2%) | 19 (8.4%) | 0.748 |
| Echinocandins & 5-flucytosine | 2 (1.4%) | 4 (4.6%) | 6 (2.7%) | 0.209 |
| Azoles & 5-flucytosine | 3 (2.2%) | 3 (3.4%) | 6 (2.7%) | 0.679 |
| Antifungal treatment duration | 98.0 (60.0 - 186.0) | 89.0 (53.0 - 160.0) | 92.0 (56.3 - 181.8) | 0.105 |
| Duration < 6 weeks | 17 (15.6%) | 15 (19.5%) | 32 (17.2%) | 0.489 |
| Duration between 6 and 12 weeks | 22 (20.2%) | 20 (26.0%) | 42 (22.6%) | 0.352 |
| Duration > 12 weeks | 70 (64.2%) | 42 (54.5%) | 112 (60.2%) | 0.184 |
| Antifungal-impregnated cement spacer | 10 (6.5%) | 2 (2.1%) | 12 (4.8%) | 0.137 |
| **Percentage of total treatment time** |  |  |  |  |
| Azoles | 100.0 (87.3 - 100.0) | 97.9 (80.9 - 100.0) | 100.0 (85.5 - 100.0) | 0.078 |
| Echinocandins | 23.3 (8.7 - 100.0) | 21.7 (10.5 - 94.6) | 23.3 (9.2 - 100.0) | 0.889 |
| Azoles & Echinocandins | 11.8 (6.4 - 14.0) | 13.4 (11.6 - 17.1) | 12.8 (8.0 - 14.0) | 0.604 |
| Amphotericin B | 13.2 (7.8 - 14.5) | 31.2 (18.9 - 48.1) | 14.5 (10.5 - 28.1) | 0.066 |
| Echinocandins & 5-flucytosine | 75.7 (63.5 - 87.8) | 19.3 (16.2 - 40.4) | 35.9 (18.7 - 87.8) | 0.348 |
| Azoles & 5-flucytosine | 74.2 (65.3 - 87.1) | 21.6 (20.3 - 41.3) | 58.7 (30.3 - 70.9) | 0.200 |
| Concomitant antibiotic therapy | 114 (74.0%) | 79 (82.3%) | 193 (77.2%) | 0.130 |
| Concomitant antibiotic treatment duration | 45.0 (19.5 - 84.0) | 56.0 (42.0 - 84.0) | 45.0 (30.3 - 84.0) | 0.118 |
| **Outcome** |  |  |  |  |
| Cure | 105 (68.2%) | 45 (46.9%) | 150 (60.0%) | <0.001* |
| Recurrence other germs | 31 (20.1%) | 19 (19.8%) | 50 (20.0%) | 0.948 |
| Failure | 49 (31.8%) | 51 (53.1%) | 100 (40.0%) | <0.001 |
| Suppressive treatment | 8 (5.2%) | 7 (7.3%) | 15 (6.0%) | 0.497 |
| Recurrence | 18 (11.7%) | 32 (33.3%) | 50 (20.0%) | <0.001* |
| Recurrence to *Candida* spp. | 7 (4.5%) | 14 (14.6%) | 21 (8.4%) | 0.005* |
| Recurrence not documented | 6 (3.9%) | 10 (10.4%) | 16 (6.4%) | 0.040* |
| Recurrence to *Candida* spp. and other bacteria | 6 (3.9%) | 8 (8.3%) | 14 (5.6%) | 0.138 |
| Death due to infectious cause | 17 (11.0%) | 10 (10.4%) | 27 (10.8%) | 0.877 |
| Death due to other cause | 6 (3.9%) | 2 (2.1%) | 8 (3.2%) | 0.714 |
| Follow-up period | 640.0 (358.0 - 1,223.0) | 499.0 (231.3 - 1,065.0) | 579.0 (295.0 - 1,105.8) | 0.132 |
|  |  |  |  |  |

^1^ Median (IQR1 - IQR3); n (%)

^2^ Pearson's Chi-squared test; Fisher's exact test; Wilcoxon rank sum exact test

*Statistically significant

Table S6. DAIR *versus* One-stage exchange

|  | DAIR  N = 96^1^ | One-Step Surgery  N = 76^1^ | Overall  N = 172^1^ | P-Value^2^ |
| --- | --- | --- | --- | --- |
| Age (years) | 74.0 (67.8 - 78.0) | 73.0 (63.0 - 79.0) | 73.0 (66.0 - 78.0) | 0.616 |
| Age > 70 yo | 63 (65.6%) | 43 (56.6%) | 106 (61.6%) | 0.226 |
| Body Mass Index | 30.0 (26.1 - 34.9) | 30.1 (26.3 - 34.7) | 30.1 (26.3 - 34.9) | 0.771 |
| Charlson Score | 4.0 (2.0 - 5.0) | 4.0 (2.0 - 5.0) | 4.0 (2.0 - 5.0) | 0.993 |
| Male patients | 42 (43.8%) | 34 (44.7%) | 76 (44.2%) | 0.897 |
| Immunosuppression | 11 (11.5%) | 7 (9.2%) | 18 (10.5%) | 0.632 |
| Immunosuppressive treatments | 12 (13.3%) | 4 (5.3%) | 16 (9.6%) | 0.079 |
| Diabetes | 32 (33.3%) | 19 (25.0%) | 51 (29.7%) | 0.235 |
| **Localization of prosthesis** |  |  |  |  |
| Hip | 54 (56.3%) | 37 (48.7%) | 91 (52.9%) | 0.324 |
| Knee | 39 (40.6%) | 36 (47.4%) | 75 (43.6%) | 0.376 |
| Other | 3 (3.1%) | 3 (3.9%) | 6 (3.5%) | >0.999 |
| Number of previous surgeries | 4.0 (2.0 - 5.5) | 3.0 (2.0 - 5.0) | 3.0 (2.0 - 5.0) | 0.031* |
| Number of previous surgeries due to infection | 2.0 (1.0 - 3.0) | 1.0 (0.0 - 2.0) | 1.0 (0.0 - 2.0) | <0.001* |
| Time between previous surgery and index infection | 33.0 (20.0 - 77.5) | 130.0 (30.0 - 680.5) | 42.0 (21.3 - 315.8) | <0.001* |
| Previous Surgery < 1 month | 46 (48.4%) | 19 (26.8%) | 65 (39.2%) | 0.005* |
| Previous Surgery < 3 months | 73 (76.8%) | 33 (46.5%) | 106 (63.9%) | <0.001* |
| Previous infection | 80 (83.3%) | 51 (67.1%) | 131 (76.2%) | 0.013* |
| Previous infection due to *Candida* spp. | 3 (3.7%) | 3 (5.8%) | 6 (4.5%) | 0.678 |
| **Microbiology analysis of previous infections** |  |  |  |  |
| Mono-bacterial infection | 30 (40.0%) | 23 (48.9%) | 53 (43.4%) | 0.333 |
| *Staphylococcus* sp. | 55 (73.3%) | 35 (74.5%) | 90 (73.8%) | 0.890 |
| *Staphylococcus aureus* | 22 (29.3%) | 17 (36.2%) | 39 (32.0%) | 0.431 |
| Coagulase-negative staphylococci | 36 (48.0%) | 23 (48.9%) | 59 (48.4%) | 0.920 |
| *Streptococcus* sp | 1 (1.1%) | 5 (6.9%) | 6 (3.7%) | 0.088 |
| *Enterococcus* sp. | 15 (20.0%) | 7 (14.9%) | 22 (18.0%) | 0.475 |
| *Pseudomonas aeruginosa* | 16 (21.3%) | 4 (8.5%) | 20 (16.4%) | 0.063 |
| *Enterobacterales* | 32 (42.7%) | 14 (29.8%) | 46 (37.7%) | 0.153 |
| Corynebacteria | 4 (4.4%) | 4 (5.6%) | 8 (4.9%) | 0.733 |
| Anaerobes | 9 (9.9%) | 5 (6.9%) | 14 (8.6%) | 0.505 |
| Previous antibiotic therapy | 78 (97.5%) | 47 (92.2%) | 125 (95.4%) | 0.207 |
| Number of lines of ATB | 1.0 (1.0 - 2.0) | 1.0 (1.0 - 2.0) | 1.0 (1.0 - 2.0) | 0.627 |
| Previous ATB duration | 48.0 (28.8 - 74.0) | 56.0 (42.0 - 90.0) | 51.0 (30.0 - 84.0) | 0.191 |
| **Clinical signs** |  |  |  |  |
| Fever | 17 (18.7%) | 14 (19.2%) | 31 (18.9%) | 0.936 |
| Inflammatory signs | 50 (53.2%) | 28 (38.9%) | 78 (47.0%) | 0.067 |
| Purulent discharge | 2 (3.1%) | 4 (10.8%) | 6 (5.9%) | 0.188 |
| Dehiscence | 42 (44.7%) | 14 (19.7%) | 56 (33.9%) | <0.001* |
| Fistula | 38 (40.9%) | 22 (30.1%) | 60 (36.1%) | 0.153 |
| Hematoma | 25 (27.2%) | 6 (8.7%) | 31 (19.3%) | 0.003 |
| **Biological analysis** |  |  |  |  |
| Leucocyte count (G/L) | 7.5 (5.9 - 8.8) | 8.0 (6.8 - 9.1) | 7.6 (6.0 - 9.0) | 0.302 |
| Neutrophil count (G/L) | 5.1 (3.5 - 6.3) | 5.4 (3.7 - 6.3) | 5.2 (3.6 - 6.3) | 0.569 |
| C-Reactive Protein level (mg/L) | 43.0 (14.7 - 80.6) | 43.4 (14.9 - 77.0) | 43.0 (14.7 - 80.6) | 0.766 |
| ESR (mm/h) | 68.0 (32.5 - 83.5) | 36.5 (28.3 - 82.3) | 65.0 (29.5 - 84.5) | 0.888 |
| Albumin level (g/L) | 31.0 (22.0 - 35.0) | 29.6 (23.0 - 36.0) | 30.8 (22.3 - 35.8) | 0.863 |
| **Radiographic evidence of infection** |  |  |  |  |
| X-ray | 39 (43.3%) | 22 (36.7%) | 61 (40.7%) | 0.415 |
| CT scan | 10 (34.5%) | 7 (24.1%) | 17 (29.3%) | 0.387 |
| Scintigraphy | 0 (0.0%) | 1 (4.0%) | 1 (2.0%) | >0.999 |
| Loosening | 2 (2.9%) | 16 (28.1%) | 18 (14.4%) | <0.001* |
| Abscess | 8 (12.3%) | 6 (10.5%) | 14 (11.5%) | 0.758 |
| TTE/TOE | 10 (15.9%) | 4 (8.9%) | 14 (13.0%) | 0.287 |
| Signs of endocarditis | 1 (2.0%) | 0 (0.0%) | 1 (1.1%) | >0.999 |
| **Microbiology analysis of index infection** |  |  |  |  |
| Positive blood culture | 13 (13.7%) | 10 (14.1%) | 23 (13.9%) | 0.941 |
| Pluri-microbial | 55 (57.3%) | 29 (38.7%) | 84 (49.1%) | 0.016 |
| Only due to *Candida* spp. | 35 (36.5%) | 40 (52.6%) | 75 (43.6%) | 0.034* |
| *C. albicans* | 58 (60.4%) | 37 (48.7%) | 95 (55.2%) | 0.124 |
| *C. parapsilosis* | 24 (25.0%) | 29 (38.2%) | 53 (30.8%) | 0.063 |
| Other *Candida* spp. | 19 (19.8%) | 9 (11.8%) | 28 (16.3%) | 0.161 |
| **Co-infection with bacteria** | 61 (63.5%) | 36 (47.4%) | 97 (56.4%) | 0.034* |
| *Staphylococcus* sp. | 34 (37.8%) | 17 (25.0%) | 51 (32.3%) | 0.089 |
| *S. aureus* | 7 (7.8%) | 6 (8.8%) | 13 (8.2%) | 0.813 |
| Coagulase-negative staphylococci | 29 (32.2%) | 12 (17.6%) | 41 (25.9%) | 0.039* |
| *Enterococcus* sp | 10 (11.1%) | 4 (5.9%) | 14 (8.9%) | 0.252 |
| *Enterobacterales* | 20 (22.2%) | 6 (8.8%) | 26 (16.5%) | 0.025* |
| *E. coli* | 6 (6.7%) | 1 (1.5%) | 7 (4.4%) | 0.240 |
| *K. pneumoniae* | 4 (4.4%) | 2 (2.9%) | 6 (3.8%) | 0.700 |
| *Enterobacter* sp. | 9 (10.0%) | 1 (1.5%) | 10 (6.3%) | 0.044* |
| *Pseudomonas aeruginosa* | 4 (4.4%) | 3 (4.4%) | 7 (4.4%) | >0.999 |
| Corynebacteria | 3 (3.3%) | 6 (8.8%) | 9 (5.7%) | 0.175 |
| Anaerobes | 4 (4.4%) | 2 (2.9%) | 6 (3.8%) | 0.700 |
| **Antifungal susceptibility testing** |  |  |  |  |
| Resistance to Fluconazole | 10 (11.5%) | 5 (7.9%) | 15 (10.0%) | 0.473 |
| Resistance to Voriconazole | 7 (8.9%) | 1 (1.8%) | 8 (6.0%) | 0.140 |
| Resistance to Echinocandins | 11 (14.5%) | 13 (22.8%) | 24 (18.0%) | 0.216 |
| **Antifungal treatments** |  |  |  |  |
| Number of lines | 1.0 (1.0 - 2.0) | 1.0 (1.0 - 2.0) | 1.0 (1.0 - 2.0) | 0.652 |
| Antibiofilm > 1 week | 37 (42.5%) | 16 (24.6%) | 53 (34.9%) | 0.022* |
| Azoles | 73 (83.9%) | 56 (86.2%) | 129 (84.9%) | 0.702 |
| Echinocandins | 37 (42.5%) | 22 (33.8%) | 59 (38.8%) | 0.277 |
| Azoles & Echinocandins | 4 (4.6%) | 4 (6.2%) | 8 (5.3%) | 0.725 |
| Amphotericin B | 8 (9.2%) | 2 (3.1%) | 10 (6.6%) | 0.190 |
| Azoles & 5-flucytosine | 3 (3.4%) | 3 (4.6%) | 6 (3.9%) | >0.999 |
| Antifungal treatment duration | 89.0 (53.0 - 160.0) | 111.5 (58.0 - 181.3) | 92.0 (53.0 - 173.0) | 0.225 |
| Duration < 6 weeks | 15 (19.5%) | 8 (16.7%) | 23 (18.4%) | 0.693 |
| Duration between 6 and 12 weeks | 20 (26.0%) | 8 (16.7%) | 28 (22.4%) | 0.225 |
| Duration > 12 weeks | 42 (54.5%) | 32 (66.7%) | 74 (59.2%) | 0.180 |
| **Percentage of total treatment time** |  |  |  |  |
| Azoles | 97.9 (80.9 - 100.0) | 100.0 (90.3 - 100.0) | 100.0 (83.9 - 100.0) | 0.139 |
| Echinocandins | 21.7 (10.5 - 94.6) | 9.2 (5.0 - 68.0) | 19.0 (8.1 - 89.2) | 0.116 |
| Azoles & Echinocandins | 13.4 (11.6 - 17.1) | 11.5 (9.6 - 12.2) | 12.3 (10.4 - 13.6) | 0.343 |
| Amphothericin B | 31.2 (18.9 - 48.1) | 8.3 (5.9 - 10.8) | 21.6 (11.1 - 39.7) | 0.143 |
| Azoles & 5-flucytosine | 21.6 (20.3 - 41.3) | 74.2 (65.3 - 87.1) | 58.7 (30.3 - 70.9) | 0.200 |
| Concomitant antibiotic therapy | 79 (82.3%) | 50 (65.8%) | 129 (75.0%) | 0.013* |
| Concomitant antibiotic treatment duration | 56.0 (42.0 - 84.0) | 45.0 (21.0 - 84.0) | 46.5 (32.8 - 84.0) | 0.211 |
| **Outcome** |  |  |  |  |
| Cure | 45 (46.9%) | 51 (67.1%) | 96 (55.8%) | 0.008* |
| Failure | 51 (53.1%) | 25 (32.9%) | 76 (44.2%) | 0.008* |
| Suppressive treatment | 7 (7.3%) | 5 (6.6%) | 12 (7.0%) | 0.855 |
| Recurrence | 32 (33.3%) | 8 (10.5%) | 40 (23.3%) | <0.001* |
| Recurrence to *Candida* spp. | 14 (14.6%) | 3 (3.9%) | 17 (9.9%) | 0.020* |
| Recurrence not documented | 10 (10.4%) | 2 (2.6%) | 12 (7.0%) | 0.047* |
| Recurrence to *Candida* spp. and other bacteria | 8 (8.3%) | 4 (5.3%) | 12 (7.0%) | 0.433 |
| Death due to infectious cause | 10 (10.4%) | 11 (14.5%) | 21 (12.2%) | 0.420 |
| Death due to other cause | 2 (2.1%) | 1 (1.3%) | 3 (1.7%) | >0.999 |
| Recurrence other germs | 19 (19.8%) | 14 (18.4%) | 33 (19.2%) | 0.821 |
| Follow-up period | 499.0 (231.3 - 1,065.0) | 684.0 (392.3 - 1,209.0) | 586.5 (296.8 - 1,099.8) | 0.088 |

^1^ Median (IQR1 - IQR3); n (%)

^2^ Pearson's Chi-squared test; Fisher's exact test; Wilcoxon rank sum exact test

*Statistically significant

Table S7. DAIR *versus* Two-stage exchange

|  | DAIR  N = 96^1^ | Two-Step Surgery  N = 78^1^ | Overall  N = 174^1^ | P-Value^2^ |
| --- | --- | --- | --- | --- |
| Age (years) | 74.0 (67.8 - 78.0) | 70.5 (59.3 - 79.0) | 73.0 (64.0 - 78.0) | 0.106 |
| Age > 70 yo | 63 (65.6%) | 39 (50.0%) | 102 (58.6%) | 0.037* |
| Body Mass Index | 30.0 (26.1 - 34.9) | 28.2 (24.7 - 33.3) | 29.1 (24.7 - 34.2) | 0.208 |
| Charlson Score | 4.0 (2.0 - 5.0) | 3.0 (2.0 - 5.0) | 3.0 (2.0 - 5.0) | 0.519 |
| Male patients | 42 (43.8%) | 40 (51.3%) | 82 (47.1%) | 0.322 |
| Immunosuppression | 11 (11.5%) | 10 (12.8%) | 21 (12.1%) | 0.784 |
| Immunosuppressive treatments | 12 (13.3%) | 7 (9.9%) | 19 (11.8%) | 0.498 |
| Diabetes | 32 (33.3%) | 15 (19.2%) | 47 (27.0%) | 0.037* |
| **Localization of prosthesis** |  |  |  |  |
| Hip | 54 (56.3%) | 40 (51.3%) | 94 (54.0%) | 0.513 |
| Knee | 39 (40.6%) | 36 (46.2%) | 75 (43.1%) | 0.464 |
| Other | 3 (3.1%) | 2 (2.6%) | 5 (2.9%) | >0.999 |
| Number of previous surgeries | 4.0 (2.0 - 5.5) | 3.0 (2.0 - 4.8) | 3.0 (2.0 - 5.0) | 0.016* |
| Number of previous surgeries due to infection | 2.0 (1.0 - 3.0) | 1.0 (0.0 - 2.0) | 2.0 (1.0 - 3.0) | 0.009* |
| Time between previous surgery and index infection | 33.0 (20.0 - 77.5) | 124.0 (43.8 - 580.8) | 47.0 (23.0 - 236.0) | <0.001* |
| Previous Surgery < 1 month | 46 (48.4%) | 10 (14.3%) | 56 (33.9%) | <0.001* |
| Previous Surgery < 3 months | 73 (76.8%) | 33 (47.1%) | 106 (64.2%) | <0.001* |
| Previous infection | 80 (83.3%) | 59 (75.6%) | 139 (79.9%) | 0.208 |
| Previous infection due to *Candida* spp. | 3 (3.7%) | 4 (6.8%) | 7 (5.0%) | 0.455 |
| **Microbiology analysis of previous infections** |  |  |  |  |
| Mono-bacterial infection | 30 (40.0%) | 33 (62.3%) | 63 (49.2%) | 0.013* |
| *Staphylococcus* sp. | 55 (73.3%) | 34 (64.2%) | 89 (69.5%) | 0.266 |
| *Staphylococcus aureus* | 22 (29.3%) | 14 (26.4%) | 36 (28.1%) | 0.718 |
| Coagulase-negative staphylococci | 36 (48.0%) | 26 (49.1%) | 62 (48.4%) | 0.906 |
| *Enterococcus* sp. | 15 (20.0%) | 7 (13.2%) | 22 (17.2%) | 0.316 |
| *Pseudomonas aeruginosa* | 16 (21.3%) | 4 (7.5%) | 20 (15.6%) | 0.034* |
| *Enterobacterales* | 32 (42.7%) | 11 (20.8%) | 43 (33.6%) | 0.010* |
| Corynebacteria | 4 (4.4%) | 3 (4.2%) | 7 (4.3%) | >0.999 |
| Anaerobes | 9 (9.9%) | 3 (4.2%) | 12 (7.4%) | 0.165 |
| Previous antibiotic therapy | 78 (97.5%) | 56 (98.2%) | 134 (97.8%) | >0.999 |
| Number of lines of ATB | 1.0 (1.0 - 2.0) | 1.0 (1.0 - 2.0) | 1.0 (1.0 - 2.0) | 0.527 |
| Previous ATB duration | 48.0 (28.8 - 74.0) | 42.0 (28.0 - 80.0) | 45.0 (28.0 - 78.0) | 0.730 |
| **Clinical signs** |  |  |  |  |
| Fever | 17 (18.7%) | 12 (16.0%) | 29 (17.5%) | 0.651 |
| Inflammatory signs | 50 (53.2%) | 44 (59.5%) | 94 (56.0%) | 0.417 |
| Purulent discharge | 2 (3.1%) | 1 (1.7%) | 3 (2.4%) | >0.999 |
| Dehiscence | 42 (44.7%) | 22 (29.7%) | 64 (38.1%) | 0.048* |
| Fistula | 38 (40.9%) | 22 (29.7%) | 60 (35.9%) | 0.136 |
| Hematoma | 25 (27.2%) | 6 (8.2%) | 31 (18.8%) | 0.002* |
| **Biological analysis** |  |  |  |  |
| Leucocyte count (G/L) | 7.5 (5.9 - 8.8) | 7.3 (6.2 - 9.2) | 7.4 (5.9 - 9.2) | 0.941 |
| Neutrophil count (G/L) | 5.1 (3.5 - 6.3) | 4.4 (3.7 - 7.2) | 4.7 (3.6 - 6.7) | 0.942 |
| C-Reactive Protein level (mg/L) | 43.0 (14.7 - 80.6) | 23.0 (12.0 - 49.9) | 34.1 (13.9 - 69.1) | 0.062 |
| ESR (mm/h) | 68.0 (32.5 - 83.5) | 60.0 (45.0 - 90.0) | 65.0 (42.0 - 85.0) | 0.808 |
| Albumin level (g/L) | 31.0 (22.0 - 35.0) | 31.2 (24.0 - 35.0) | 31.0 (22.8 - 35.0) | 0.920 |
| **Radiographic evidence of infection** |  |  |  |  |
| X-ray | 39 (43.3%) | 35 (51.5%) | 74 (46.8%) | 0.310 |
| CT scan | 10 (34.5%) | 10 (33.3%) | 20 (33.9%) | 0.926 |
| Scintigraphy | 0 (0.0%) | 4 (16.0%) | 4 (8.2%) | 0.110 |
| Loosening | 2 (2.9%) | 14 (29.8%) | 16 (13.9%) | <0.001* |
| Abscess | 8 (12.3%) | 10 (20.8%) | 18 (15.9%) | 0.221 |
| TTE/TOE | 10 (15.9%) | 1 (2.2%) | 11 (10.2%) | 0.024 |
| Signs of endocarditis | 1 (2.0%) | 0 (0.0%) | 1 (1.2%) | >0.999 |
| **Microbiology analysis of index infection** |  |  |  |  |
| Positive blood culture | 13 (13.7%) | 5 (6.8%) | 18 (10.7%) | 0.148 |
| Pluri-microbial | 55 (57.3%) | 36 (46.2%) | 91 (52.3%) | 0.144 |
| Only due to *Candida* spp. | 35 (36.5%) | 41 (52.6%) | 76 (43.7%) | 0.033* |
| *C. albicans* | 58 (60.4%) | 40 (51.3%) | 98 (56.3%) | 0.227 |
| *C. parapsilosis* | 24 (25.0%) | 23 (29.5%) | 47 (27.0%) | 0.507 |
| Other *Candida* spp. | 19 (19.8%) | 17 (21.8%) | 36 (20.7%) | 0.746 |
| **Co-infection with bacteria** | 61 (63.5%) | 37 (47.4%) | 98 (56.3%) | 0.033* |
| *Staphylococcus* sp. | 34 (37.8%) | 20 (26.0%) | 54 (32.3%) | 0.104 |
| *S. aureus* | 7 (7.8%) | 10 (13.0%) | 17 (10.2%) | 0.267 |
| Coagulase-negative staphylococci | 29 (32.2%) | 12 (15.6%) | 41 (24.6%) | 0.013* |
| *Enterococcus* sp | 10 (11.1%) | 4 (5.2%) | 14 (8.4%) | 0.169 |
| *Enterobacterales* | 20 (22.2%) | 10 (13.0%) | 30 (18.0%) | 0.121 |
| *E. coli* | 6 (6.7%) | 3 (3.9%) | 9 (5.4%) | 0.508 |
| *K. pneumoniae* | 4 (4.4%) | 4 (5.2%) | 8 (4.8%) | >0.999 |
| *Enterobacter* sp. | 9 (10.0%) | 1 (1.3%) | 10 (6.0%) | 0.021* |
| *Pseudomonas aeruginosa* | 4 (4.4%) | 2 (2.6%) | 6 (3.6%) | 0.688 |
| Corynebacteria | 3 (3.3%) | 4 (5.2%) | 7 (4.2%) | 0.705 |
| Anaerobes | 4 (4.4%) | 2 (2.6%) | 6 (3.6%) | 0.688 |
| **Antifungal susceptibility testing** |  |  |  |  |
| Resistance to Fluconazole | 10 (11.5%) | 5 (7.5%) | 15 (9.7%) | 0.403 |
| Resistance to Voriconazole | 7 (8.9%) | 2 (3.5%) | 9 (6.6%) | 0.303 |
| Resistance to Echinocandins | 11 (14.5%) | 4 (7.4%) | 15 (11.5%) | 0.214 |
| **Antifungal treatments** |  |  |  |  |
| Number of lines | 1.0 (1.0 - 2.0) | 1.0 (1.0 - 2.0) | 1.0 (1.0 - 2.0) | 0.358 |
| Antibiofilm > 1 week | 37 (42.5%) | 19 (26.0%) | 56 (35.0%) | 0.029* |
| Azoles | 73 (83.9%) | 62 (84.9%) | 135 (84.4%) | 0.859 |
| Echinocandins | 37 (42.5%) | 22 (30.1%) | 59 (36.9%) | 0.106 |
| Azoles & Echinocandins | 4 (4.6%) | 5 (6.8%) | 9 (5.6%) | 0.733 |
| Amphotericin B | 8 (9.2%) | 9 (12.3%) | 17 (10.6%) | 0.522 |
| Antifungal treatment duration | 89.0 (53.0 - 160.0) | 92.0 (61.0 - 193.0) | 92.0 (56.3 - 183.3) | 0.136 |
| Duration < 6 weeks | 15 (19.5%) | 9 (14.8%) | 24 (17.4%) | 0.467 |
| Duration between 6 and 12 weeks | 20 (26.0%) | 14 (23.0%) | 34 (24.6%) | 0.682 |
| Duration > 12 weeks | 42 (54.5%) | 38 (62.3%) | 80 (58.0%) | 0.360 |
| Antifungal-impregnated cement spacer | 2 (2.1%) | 9 (11.5%) | 11 (6.3%) | 0.013* |
| **Percentage of total treatment time** |  |  |  |  |
| Azoles | 97.9 (80.9 - 100.0) | 100.0 (86.5 - 100.0) | 100.0 (81.2 - 100.0) | 0.145 |
| Echinocandins | 21.7 (10.5 - 94.6) | 49.1 (15.6 - 100.0) | 25.3 (11.2 - 100.0) | 0.289 |
| Azoles & Echinocandins | 13.4 (11.6 - 17.1) | 14.0 (6.4 - 26.9) | 14.0 (8.0 - 26.6) | >0.999 |
| Amphotericin B | 31.2 (18.9 - 48.1) | 14.5 (10.5 - 18.3) | 16.3 (13.2 - 29.2) | 0.138 |
| Concomitant antibiotic therapy | 79 (82.3%) | 64 (82.1%) | 143 (82.2%) | 0.967 |
| Concomitant antibiotic treatment duration | 56.0 (42.0 - 84.0) | 43.5 (18.3 - 84.0) | 45.0 (33.8 - 84.0) | 0.180 |
| **Outcome** |  |  |  |  |
| Cure | 45 (46.9%) | 54 (69.2%) | 99 (56.9%) | 0.003* |
| Recurrence other germs | 19 (19.8%) | 17 (21.8%) | 36 (20.7%) | 0.746 |
| Failure | 51 (53.1%) | 24 (30.8%) | 75 (43.1%) | 0.003* |
| Suppressive treatment | 7 (7.3%) | 3 (3.8%) | 10 (5.7%) | 0.515 |
| Recurrence | 32 (33.3%) | 10 (12.8%) | 42 (24.1%) | 0.002* |
| Recurrence to *Candida* spp. | 14 (14.6%) | 4 (5.1%) | 18 (10.3%) | 0.042* |
| Recurrence not documented | 10 (10.4%) | 4 (5.1%) | 14 (8.0%) | 0.202 |
| Recurrence to *Candida* spp. and other bacteria | 8 (8.3%) | 2 (2.6%) | 10 (5.7%) | 0.188 |
| Death due to infectious cause | 10 (10.4%) | 6 (7.7%) | 16 (9.2%) | 0.536 |
| Death due to other cause | 2 (2.1%) | 5 (6.4%) | 7 (4.0%) | 0.245 |
| Follow-up period | 499.0 (231.3 - 1,065.0) | 564.5 (300.8 - 1,237.0) | 540.0 (257.3 - 1,089.0) | 0.411 |

^1^ Median (IQR1 - IQR3); n (%)

^2^ Pearson's Chi-squared test; Fisher's exact test; Wilcoxon rank sum exact test

*Statistically significant

Table S8. One-stage exchange *versus* Two-stage exchange

|  | One-Stage Exchange  N = 76^1^ | Two-Stage Exchange  N = 78^1^ | Overall  N = 154^1^ | P-Value^2^ |
| --- | --- | --- | --- | --- |
| Age (years) | 73.0 (63.0 - 79.0) | 70.5 (59.3 - 79.0) | 71.0 (61.0 - 79.0) | 0.300 |
| Age > 70 yo | 43 (56.6%) | 39 (50.0%) | 82 (53.2%) | 0.413 |
| Body Mass Index | 30.1 (26.3 - 34.7) | 28.2 (24.7 - 33.3) | 28.9 (25.2 - 33.9) | 0.121 |
| Charlson Score | 4.0 (2.0 - 5.0) | 3.0 (2.0 - 5.0) | 3.5 (2.0 - 5.0) | 0.555 |
| Male patients | 34 (44.7%) | 40 (51.3%) | 74 (48.1%) | 0.416 |
| Immunosuppression | 7 (9.2%) | 10 (12.8%) | 17 (11.0%) | 0.475 |
| Immunosuppressive treatments | 4 (5.3%) | 7 (9.9%) | 11 (7.5%) | 0.290 |
| Diabetes | 19 (25.0%) | 15 (19.2%) | 34 (22.1%) | 0.388 |
| **Localization of prosthesis** |  |  |  |  |
| Hip | 37 (48.7%) | 40 (51.3%) | 77 (50.0%) | 0.747 |
| Knee | 36 (47.4%) | 36 (46.2%) | 72 (46.8%) | 0.880 |
| Other | 3 (3.9%) | 2 (2.6%) | 5 (3.2%) | 0.679 |
| Number of previous surgeries | 3.0 (2.0 - 5.0) | 3.0 (2.0 - 4.8) | 3.0 (2.0 - 5.0) | 0.770 |
| Number of previous surgeries due to infection | 1.0 (0.0 - 2.0) | 1.0 (0.0 - 2.0) | 1.0 (0.0 - 2.0) | 0.278 |
| Time between previous surgery and index infection | 130.0 (30.0 - 680.5) | 124.0 (43.8 - 580.8) | 130.0 (38.0 - 587.0) | 0.767 |
| Previous Surgery < 1 month | 19 (26.8%) | 10 (14.3%) | 29 (20.6%) | 0.067 |
| Previous Surgery < 3 months | 33 (46.5%) | 33 (47.1%) | 66 (46.8%) | 0.937 |
| Previous infection | 51 (67.1%) | 59 (75.6%) | 110 (71.4%) | 0.241 |
| Previous infection due to *Candida* spp. | 3 (5.8%) | 4 (6.8%) | 7 (6.3%) | >0.999 |
| **Microbiology analysis of previous infections** |  |  |  |  |
| Mono-bacterial infection | 23 (48.9%) | 33 (62.3%) | 56 (56.0%) | 0.180 |
| *Staphylococcus* sp. | 35 (74.5%) | 34 (64.2%) | 69 (69.0%) | 0.266 |
| *Staphylococcus aureus* | 17 (36.2%) | 14 (26.4%) | 31 (31.0%) | 0.292 |
| Coagulase-negative staphylococci | 23 (48.9%) | 26 (49.1%) | 49 (49.0%) | 0.990 |
| *Streptococcus* sp | 5 (6.9%) | 3 (4.2%) | 8 (5.6%) | 0.719 |
| *Enterococcus* sp. | 7 (14.9%) | 7 (13.2%) | 14 (14.0%) | 0.808 |
| *Pseudomonas aeruginosa* | 4 (8.5%) | 4 (7.5%) | 8 (8.0%) | >0.999 |
| *Enterobacterales* | 14 (29.8%) | 11 (20.8%) | 25 (25.0%) | 0.298 |
| Corynebacteria | 4 (5.6%) | 3 (4.2%) | 7 (4.9%) | >0.999 |
| Anaerobes | 5 (6.9%) | 3 (4.2%) | 8 (5.6%) | 0.719 |
| Previous antibiotic therapy | 47 (92.2%) | 56 (98.2%) | 103 (95.4%) | 0.186 |
| Number of lines of ATB | 1.0 (1.0 - 2.0) | 1.0 (1.0 - 2.0) | 1.0 (1.0 - 2.0) | 0.942 |
| Previous ATB duration | 56.0 (42.0 - 90.0) | 42.0 (28.0 - 80.0) | 47.0 (29.0 - 87.0) | 0.124 |
| **Clinical signs** |  |  |  |  |
| Fever | 14 (19.2%) | 12 (16.0%) | 26 (17.6%) | 0.611 |
| Inflammatory signs | 28 (38.9%) | 44 (59.5%) | 72 (49.3%) | 0.013* |
| Purulent discharge | 4 (10.8%) | 1 (1.7%) | 5 (5.2%) | 0.068 |
| Dehiscence | 14 (19.7%) | 22 (29.7%) | 36 (24.8%) | 0.163 |
| Fistula | 22 (30.1%) | 22 (29.7%) | 44 (29.9%) | 0.957 |
| Hematoma | 6 (8.7%) | 6 (8.2%) | 12 (8.5%) | 0.919 |
| **Biological analysis** |  |  |  |  |
| Leucocyte count (G/L) | 8.0 (6.8 - 9.1) | 7.3 (6.2 - 9.2) | 7.6 (6.3 - 9.2) | 0.322 |
| Neutrophil count (G/L) | 5.4 (3.7 - 6.3) | 4.4 (3.7 - 7.2) | 5.0 (3.7 - 6.6) | 0.617 |
| C-Reactive Protein level (mg/L) | 43.4 (14.9 - 77.0) | 23.0 (12.0 - 49.9) | 29.0 (13.6 - 66.0) | 0.180 |
| ESR (mm/h) | 36.5 (28.3 - 82.3) | 60.0 (45.0 - 90.0) | 59.0 (39.5 - 92.5) | 0.234 |
| Albumin level (g/L) | 29.6 (23.0 - 36.0) | 31.2 (24.0 - 35.0) | 30.0 (23.0 - 36.0) | 0.971 |
| **Radiographic evidence of infection** |  |  |  |  |
| X-ray | 22 (36.7%) | 35 (51.5%) | 57 (44.5%) | 0.093 |
| CT scan | 7 (24.1%) | 10 (33.3%) | 17 (28.8%) | 0.436 |
| Scintigraphy | 1 (4.0%) | 4 (16.0%) | 5 (10.0%) | 0.349 |
| Loosening | 16 (28.1%) | 14 (29.8%) | 30 (28.8%) | 0.847 |
| Abscess | 6 (10.5%) | 10 (20.8%) | 16 (15.2%) | 0.143 |
| **Microbiology analysis of index infection** |  |  |  |  |
| Positive blood culture | 10 (14.1%) | 5 (6.8%) | 15 (10.3%) | 0.148 |
| Pluri-microbial | 29 (38.7%) | 36 (46.2%) | 65 (42.5%) | 0.349 |
| Only due to *Candida* spp. | 40 (52.6%) | 41 (52.6%) | 81 (52.6%) | 0.993 |
| *C. albicans* | 37 (48.7%) | 40 (51.3%) | 77 (50.0%) | 0.747 |
| *C. parapsilosis* | 29 (38.2%) | 23 (29.5%) | 52 (33.8%) | 0.255 |
| Other *Candida* spp. | 9 (11.8%) | 17 (21.8%) | 26 (16.9%) | 0.099 |
| **Co-infection with bacteria** | 36 (47.4%) | 37 (47.4%) | 73 (47.4%) | 0.993 |
| *Staphylococcus* sp. | 17 (25.0%) | 20 (26.0%) | 37 (25.5%) | 0.893 |
| *S. aureus* | 6 (8.8%) | 10 (13.0%) | 16 (11.0%) | 0.425 |
| Coagulase-negative staphylococci | 12 (17.6%) | 12 (15.6%) | 24 (16.6%) | 0.739 |
| *Enterococcus* sp | 4 (5.9%) | 4 (5.2%) | 8 (5.5%) | >0.999 |
| *Enterobacterales* | 6 (8.8%) | 10 (13.0%) | 16 (11.0%) | 0.425 |
| *Pseudomonas aeruginosa* | 3 (4.4%) | 2 (2.6%) | 5 (3.4%) | 0.666 |
| Corynebacteria | 6 (8.8%) | 4 (5.2%) | 10 (6.9%) | 0.516 |
| **Antifungal susceptibility testing** |  |  |  |  |
| Resistance to Fluconazole | 5 (7.9%) | 5 (7.5%) | 10 (7.7%) | >0.999 |
| Resistance to Echinocandins | 13 (22.8%) | 4 (7.4%) | 17 (15.3%) | 0.024* |
| **Antifungal treatments** |  |  |  |  |
| Number of lines | 1.0 (1.0 - 2.0) | 1.0 (1.0 - 2.0) | 1.0 (1.0 - 2.0) | 0.706 |
| Antibiofilm > 1 week | 16 (24.6%) | 19 (26.0%) | 35 (25.4%) | 0.849 |
| Azoles | 56 (86.2%) | 62 (84.9%) | 118 (85.5%) | 0.839 |
| Echinocandins | 22 (33.8%) | 22 (30.1%) | 44 (31.9%) | 0.641 |
| Azoles & Echinocandins | 4 (6.2%) | 5 (6.8%) | 9 (6.5%) | >0.999 |
| Amphotericin B | 2 (3.1%) | 9 (12.3%) | 11 (8.0%) | 0.045 |
| Antifungal treatment duration | 111.5 (58.0 - 181.3) | 92.0 (61.0 - 193.0) | 98.0 (60.0 - 186.0) | 0.793 |
| Duration < 6 weeks | 8 (16.7%) | 9 (14.8%) | 17 (15.6%) | 0.785 |
| Duration between 6 and 12 weeks | 8 (16.7%) | 14 (23.0%) | 22 (20.2%) | 0.417 |
| Duration > 12 weeks | 32 (66.7%) | 38 (62.3%) | 70 (64.2%) | 0.636 |
| Antifungal-impregnated cement spacer | 1 (1.3%) | 9 (11.5%) | 10 (6.5%) | 0.018 |
| **Percentage of total treatment time** |  |  |  |  |
| Azoles | 100.0 (90.3 - 100.0) | 100.0 (86.5 - 100.0) | 100.0 (87.3 - 100.0) | 0.871 |
| Echinocandins | 9.2 (5.0 - 68.0) | 49.1 (15.6 - 100.0) | 23.3 (8.7 - 100.0) | 0.041 |
| Azoles & Echinocandins | 11.5 (9.6 - 12.2) | 14.0 (6.4 - 26.9) | 11.8 (6.4 - 14.0) | 0.556 |
| Amphotericin B | 8.3 (5.9 - 10.8) | 14.5 (10.5 - 18.3) | 13.2 (7.8 - 14.5) | 0.222 |
| Concomitant antibiotic therapy | 50 (65.8%) | 64 (82.1%) | 114 (74.0%) | 0.021* |
| Concomitant antibiotic treatment duration | 45.0 (21.0 - 84.0) | 43.5 (18.3 - 84.0) | 45.0 (19.5 - 84.0) | 0.946 |
| **Outcome** |  |  |  |  |
| Cure | 51 (67.1%) | 54 (69.2%) | 105 (68.2%) | 0.777 |
| Recurrence other germs | 14 (18.4%) | 17 (21.8%) | 31 (20.1%) | 0.602 |
| Failure | 25 (32.9%) | 24 (30.8%) | 49 (31.8%) | 0.777 |
| Suppressive treatment | 5 (6.6%) | 3 (3.8%) | 8 (5.2%) | 0.492 |
| Recurrence | 8 (10.5%) | 10 (12.8%) | 18 (11.7%) | 0.658 |
| Recurrence to *Candida* spp. | 3 (3.9%) | 4 (5.1%) | 7 (4.5%) | >0.999 |
| Recurrence not documented | 2 (2.6%) | 4 (5.1%) | 6 (3.9%) | 0.681 |
| Recurrence to *Candida* spp. and other bacteria | 4 (5.3%) | 2 (2.6%) | 6 (3.9%) | 0.439 |
| Death due to infectious cause | 11 (14.5%) | 6 (7.7%) | 17 (11.0%) | 0.179 |
| Death due to other cause | 1 (1.3%) | 5 (6.4%) | 6 (3.9%) | 0.210 |
| Follow-up period | 684.0 (392.3 - 1,209.0) | 564.5 (300.8 - 1,237.0) | 640.0 (358.0 - 1,223.0) | 0.482 |

^1^ Median (IQR1 - IQR3); n (%)

^2^ Pearson's Chi-squared test; Fisher's exact test; Wilcoxon rank sum exact test

*Statistically significant

## Analysis on *Candida* PJI depending on antifungal treatment duration

Table S9. Patient's characteristics according to antifungal duration (< 6 weeks *versus* 6-12 weeks)

|  | 6 to 12 Weeks  N = 44^1^ | < 6 Weeks  N = 36^1^ | Overall  N = 80^1^ | P-value^2^ |
| --- | --- | --- | --- | --- |
| Age (year) | 75.0 (66.8 - 82.0) | 75.0 (68.0 - 80.3) | 75.0 (67.8 - 81.0) | 0.900 |
| Age > 70 yo | 26 (59.1%) | 25 (69.4%) | 51 (63.8%) | 0.338 |
| Body Mass Index | 30.8 (26.5 - 34.7) | 32.3 (26.7 - 36.4) | 30.9 (26.6 - 35.4) | 0.580 |
| Charlson score | 4.0 (2.0 - 5.0) | 4.0 (3.0 - 6.0) | 4.0 (2.5 - 6.0) | 0.219 |
| Male patients | 20 (45.5%) | 11 (30.6%) | 31 (38.8%) | 0.174 |
| Immunosuppression | 2 (4.5%) | 4 (11.1%) | 6 (7.5%) | 0.401 |
| Immunosuppressive treatments | 3 (8.1%) | 4 (12.1%) | 7 (10.0%) | 0.699 |
| Diabetes | 13 (29.5%) | 10 (27.8%) | 23 (28.8%) | 0.862 |
| **Localization of prosthesis** |  |  |  |  |
| Hip | 26 (59.1%) | 22 (61.1%) | 48 (60.0%) | 0.854 |
| Knee | 16 (36.4%) | 13 (36.1%) | 29 (36.3%) | 0.981 |
| Other | 2 (4.5%) | 1 (2.8%) | 3 (3.8%) | >0.999 |
| Number of previous surgeries | 3.0 (2.0 - 5.0) | 2.0 (1.8 - 3.3) | 3.0 (2.0 - 4.0) | 0.013* |
| Number of previous surgeries due to infection | 1.0 (1.0 - 2.0) | 1.0 (0.0 - 2.0) | 1.0 (1.0 - 2.0) | 0.056 |
| Time between previous surgery and index infection | 105.0 (21.0 - 598.5) | 48.0 (26.0 - 279.0) | 60.0 (21.0 - 471.0) | 0.502 |
| Previous Surgery < 1 month | 17 (39.5%) | 10 (30.3%) | 27 (35.5%) | 0.405 |
| Previous Surgery < 3 months | 21 (48.8%) | 20 (60.6%) | 41 (53.9%) | 0.308 |
| Previous infection | 37 (84.1%) | 22 (61.1%) | 59 (73.8%) | 0.020* |
| Previous infection due to *Candida* spp. | 3 (7.9%) | 3 (13.0%) | 6 (9.8%) | 0.664 |
| **Microbiology analysis of previous infections** |  |  |  |  |
| Mono-bacterial infection | 18 (54.5%) | 11 (55.0%) | 29 (54.7%) | 0.974 |
| *Staphylococcus* sp. | 16 (48.5%) | 16 (80.0%) | 32 (60.4%) | 0.023* |
| *Staphylococcus aureus* | 5 (15.2%) | 6 (30.0%) | 11 (20.8%) | 0.296 |
| Coagulase-negative staphylococci | 13 (39.4%) | 10 (50.0%) | 23 (43.4%) | 0.450 |
| *Streptococcus* sp | 4 (10.0%) | 3 (8.8%) | 7 (9.5%) | >0.999 |
| *Enterococcus* sp. | 5 (15.2%) | 3 (15.0%) | 8 (15.1%) | >0.999 |
| *Pseudomonas aeruginosa* | 7 (21.2%) | 4 (20.0%) | 11 (20.8%) | >0.999 |
| Enterobacterales | 10 (30.3%) | 8 (40.0%) | 18 (34.0%) | 0.470 |
| Corynebacteria | 1 (2.5%) | 1 (2.9%) | 2 (2.7%) | >0.999 |
| Anaerobes | 3 (7.5%) | 2 (5.9%) | 5 (6.8%) | >0.999 |
| Previous antibiotic therapy | 35 (94.6%) | 21 (95.5%) | 56 (94.9%) | >0.999 |
| Number of lines of ATB | 1.0 (1.0 - 2.0) | 1.0 (1.0 - 2.5) | 1.0 (1.0 - 2.0) | 0.694 |
| Previous ATB duration | 49.0 (43.0 - 81.5) | 39.5 (28.5 - 61.0) | 47.0 (30.0 - 71.0) | 0.151 |
| **Clinical signs** |  |  |  |  |
| Fever | 4 (9.3%) | 8 (22.2%) | 12 (15.2%) | 0.111 |
| Inflammatory signs | 15 (35.7%) | 21 (60.0%) | 36 (46.8%) | 0.033* |
| Purulent discharge | 0 (0.0%) | 4 (17.4%) | 4 (7.7%) | 0.033 |
| Dehiscence | 14 (32.6%) | 11 (33.3%) | 25 (32.9%) | 0.943 |
| Fistula | 16 (38.1%) | 12 (35.3%) | 28 (36.8%) | 0.801 |
| Hematoma | 8 (19.0%) | 6 (18.8%) | 14 (18.9%) | 0.974 |
| **Biological analysis** |  |  |  |  |
| Leucocyte count (G/L) | 7.2 (5.8 - 8.6) | 8.0 (6.9 - 10.8) | 7.5 (6.2 - 9.2) | 0.069 |
| Neutrophil count (G/L) | 4.5 (3.8 - 6.0) | 5.5 (3.9 - 6.8) | 5.0 (3.8 - 6.4) | 0.233 |
| C-Reactive Protein level (mg/L) | 25.2 (8.1 - 54.4) | 47.0 (17.1 - 92.6) | 29.6 (12.0 - 70.0) | 0.060 |
| ESR (mm/h) | 55.5 (17.0 - 85.0) | 35.0 (34.0 - 41.0) | 41.0 (24.0 - 80.0) | 0.859 |
| Albumin level (g/L) | 33.0 (28.5 - 36.0) | 23.5 (20.0 - 31.0) | 30.0 (22.0 - 35.0) | 0.009* |
| **Radiographic evidence of infection** |  |  |  |  |
| X-ray | 19 (47.5%) | 12 (41.4%) | 31 (44.9%) | 0.614 |
| CT scan | 2 (16.7%) | 5 (38.5%) | 7 (28.0%) | 0.378 |
| Scintigraphy | 2 (20.0%) | 1 (8.3%) | 3 (13.6%) | 0.571 |
| Loosening | 5 (17.9%) | 3 (11.5%) | 8 (14.8%) | 0.706 |
| Abscess | 2 (7.7%) | 5 (20.0%) | 7 (13.7%) | 0.248 |
| TTE/TOE | 3 (10.3%) | 5 (22.7%) | 8 (15.7%) | 0.268 |
| Signs of endocarditis | 0 (0.0%) | 1 (5.0%) | 1 (2.8%) | >0.999 |
| **Type of surgery** |  |  |  |  |
| DAIR | 20 (45.5%) | 15 (42.9%) | 35 (44.3%) | 0.817 |
| Exchange surgery | 22 (50.0%) | 17 (48.6%) | 39 (49.4%) | 0.900 |
| One-stage exchange | 8 (18.2%) | 8 (22.9%) | 16 (20.3%) | 0.608 |
| Two-stage exchange | 14 (31.8%) | 9 (25.7%) | 23 (29.1%) | 0.553 |
| Other type of surgery | 2 (4.5%) | 3 (8.6%) | 5 (6.3%) | 0.650 |
| **Microbiology analysis of index infection** |  |  |  |  |
| Positive blood culture | 3 (7.0%) | 7 (20.0%) | 10 (12.8%) | 0.103 |
| Pluri-microbial | 22 (50.0%) | 23 (63.9%) | 45 (56.3%) | 0.213 |
| Only due to *Candida* spp. | 21 (47.7%) | 10 (27.8%) | 31 (38.8%) | 0.068 |
| *C. albicans* | 28 (63.6%) | 18 (50.0%) | 46 (57.5%) | 0.220 |
| *C. parapsilosis* | 12 (27.3%) | 10 (27.8%) | 22 (27.5%) | 0.960 |
| Other *Candida* spp. | 7 (15.9%) | 9 (25.0%) | 16 (20.0%) | 0.312 |
| **Co-infection with bacteria** | 23 (52.3%) | 26 (72.2%) | 49 (61.3%) | 0.068 |
| *Staphylococcus* sp. | 9 (22.5%) | 19 (54.3%) | 28 (37.3%) | 0.005* |
| *S. aureus* | 2 (5.0%) | 9 (25.7%) | 11 (14.7%) | 0.011* |
| Coagulase-negative staphylococci | 7 (17.5%) | 13 (37.1%) | 20 (26.7%) | 0.055 |
| *Enterococcus* sp | 5 (12.5%) | 3 (8.6%) | 8 (10.7%) | 0.716 |
| *Streptococcus* sp. | 1 (2.5%) | 1 (2.9%) | 2 (2.7%) | >0.999 |
| Enterobacterales | 7 (17.5%) | 7 (20.0%) | 14 (18.7%) | 0.782 |
| *E. coli* | 0 (0.0%) | 4 (11.4%) | 4 (5.3%) | 0.043* |
| *K. pneumoniae* | 4 (10.0%) | 1 (2.9%) | 5 (6.7%) | 0.364 |
| *Enterobacter* sp. | 1 (2.5%) | 2 (5.7%) | 3 (4.0%) | 0.596 |
| *Pseudomonas aeruginosa* | 2 (5.0%) | 2 (5.7%) | 4 (5.3%) | >0.999 |
| Corynebacteria | 1 (2.5%) | 2 (5.7%) | 3 (4.0%) | 0.596 |
| Anaerobes | 3 (7.5%) | 1 (2.9%) | 4 (5.3%) | 0.618 |
| **Antifungal susceptibility testing** |  |  |  |  |
| Resistance to Fluconazole | 3 (7.7%) | 1 (3.6%) | 4 (6.0%) | 0.635 |
| Resistance to Voriconazole | 2 (6.1%) | 0 (0.0%) | 2 (3.4%) | 0.501 |
| Resistance to Posaconazole | 1 (7.1%) | 0 (0.0%) | 1 (4.8%) | >0.999 |
| Resistance to Echinocandins | 2 (6.5%) | 4 (16.7%) | 6 (10.9%) | 0.387 |
| Resistance to 5-fluorocytosine | 3 (16.7%) | 0 (0.0%) | 3 (11.1%) | 0.529 |
| **Antifungal treatments** |  |  |  |  |
| Number of lines | 1.0 (1.0 - 2.0) | 1.0 (1.0 - 1.0) | 1.0 (1.0 - 1.3) | <0.001* |
| Antibiofilm > 1 week | 13 (29.5%) | 9 (25.0%) | 22 (27.5%) | 0.651 |
| Azoles | 36 (81.8%) | 25 (69.4%) | 61 (76.3%) | 0.196 |
| Echinocandins | 13 (29.5%) | 10 (27.8%) | 23 (28.8%) | 0.862 |
| Azoles & Echinocandins | 5 (11.4%) | 0 (0.0%) | 5 (6.3%) | 0.061 |
| Amphotericin B | 6 (13.6%) | 0 (0.0%) | 6 (7.5%) | 0.030* |
| Echinocandins & 5-flucytosine | 0 (0.0%) | 1 (2.8%) | 1 (1.3%) | 0.450 |
| Azoles & 5-flucytosine | 3 (6.8%) | 0 (0.0%) | 3 (3.8%) | 0.248 |
| Antifungal treatment duration | 60.5 (53.0 - 65.5) | 29.5 (14.0 - 37.3) | 45.5 (30.0 - 61.0) | <0.001* |
| Antifungal-impregnated cement spacer | 4 (9.1%) | 0 (0.0%) | 4 (5.0%) | 0.123 |
| **Percentage of total treatment time** |  |  |  |  |
| Azoles | 100.0 (75.8 - 100.0) | 100.0 (100.0 - 100.0) | 100.0 (85.5 - 100.0) | 0.005* |
| Echinocandins | 48.8 (19.0 - 100.0) | 100.0 (100.0 - 100.0) | 100.0 (41.0 - 100.0) | 0.037* |
| Azoles & Echinocandins | 26.6 (14.0 - 26.9) | NA (NA - NA) | 26.6 (14.0 - 26.9) |  |
| Amphotericin B | 32.4 (27.5 - 48.1) | NA (NA - NA) | 32.4 (27.5 - 48.1) |  |
| Echinocandins & 5-flucytosine | NA (NA - NA) | 100.0 (100.0 - 100.0) | 100.0 (100.0 - 100.0) |  |
| Azoles & 5-flucytosine | 56.4 (37.7 - 78.2) | NA (NA - NA) | 56.4 (37.7 - 78.2) |  |
| Concomitant antibiotic therapy | 32 (72.7%) | 33 (91.7%) | 65 (81.3%) | 0.031* |
| Concomitant antibiotic treatment duration | 56.0 (42.0 - 84.0) | 42.0 (25.0 - 45.0) | 42.0 (38.0 - 76.0) | 0.016* |
| **Outcome** |  |  |  |  |
| Cure | 29 (65.9%) | 18 (50.0%) | 47 (58.8%) | 0.150 |
| Recurrence other germs | 12 (27.3%) | 5 (13.9%) | 17 (21.3%) | 0.145 |
| Failure | 15 (34.1%) | 18 (50.0%) | 33 (41.3%) | 0.150 |
| Suppressive treatment | 0 (0.0%) | 0 (0.0%) | 0 (0.0%) |  |
| Recurrence | 8 (18.2%) | 5 (13.9%) | 13 (16.3%) | 0.605 |
| Recurrence to *Candida* spp. | 5 (11.4%) | 3 (8.3%) | 8 (10.0%) | 0.724 |
| Recurrence not documented | 1 (2.3%) | 1 (2.8%) | 2 (2.5%) | >0.999 |
| Recurrence to *Candida* spp. and other bacteria | 2 (4.5%) | 1 (2.8%) | 3 (3.8%) | >0.999 |
| Death due to infectious cause | 5 (11.4%) | 9 (25.0%) | 14 (17.5%) | 0.110 |
| Death due to other cause | 2 (4.5%) | 4 (11.1%) | 6 (7.5%) | 0.401 |
| Follow-up period | 746.0 (252.0 - 1,504.0) | 359.0 (115.5 - 732.5) | 426.0 (188.0 - 1,298.5) | 0.074 |

^1^ Median (IQR1 - IQR3); n (%)

^2^ Pearson's Chi-squared test; Fisher's exact test; Wilcoxon rank sum exact test

*Statistically significant

Table S10. Patients' characteristics according to antifungal duration (< 6 weeks *versus* > 12 weeks)

|  | | > 12 Weeks  N = 116^1^ | | < 6 Weeks  N = 36^1^ | | Overall  N = 152^1^ | | P-value^2^ | |
| --- | --- | --- | --- | --- | --- | --- | --- | --- | --- |
| Age (year) | | 71.0 (61.0 - 77.0) | | 75.0 (68.0 - 80.3) | | 72.0 (63.8 - 79.0) | | 0.017* | |
| Age > 70 yo | | 62 (53.4%) | | 25 (69.4%) | | 87 (57.2%) | | 0.090 | |
| Body Mass Index | | 29.4 (24.5 - 34.4) | | 32.3 (26.7 - 36.4) | | 29.9 (24.8 - 35.0) | | 0.206 | |
| Charlson score | | 3.0 (2.0 - 5.0) | | 4.0 (3.0 - 6.0) | | 4.0 (2.0 - 5.0) | | 0.016* | |
| Male patients | | 63 (54.3%) | | 11 (30.6%) | | 74 (48.7%) | | 0.013* | |
| Immunosuppression | | 16 (13.8%) | | 4 (11.1%) | | 20 (13.2%) | | 0.785 | |
| Immunosuppressive treatments | | 12 (10.6%) | | 4 (12.1%) | | 16 (11.0%) | | 0.759 | |
| Diabetes | | 33 (28.4%) | | 10 (27.8%) | | 43 (28.3%) | | 0.938 | |
| Hip | | 58 (50.0%) | | 22 (61.1%) | | 80 (52.6%) | | 0.243 | |
| **Localization of prosthesis** |  | |  | |  | |  | |  |
| Knee | | 53 (45.7%) | | 13 (36.1%) | | 66 (43.4%) | | 0.311 | |
| Other | | 5 (4.3%) | | 1 (2.8%) | | 6 (3.9%) | | >0.999 | |
| Number of previous surgeries | | 3.0 (2.0 - 5.0) | | 2.0 (1.8 - 3.3) | | 3.0 (2.0 - 5.0) | | 0.014* | |
| Number of previous surgeries due to infection | | 1.0 (0.0 - 2.0) | | 1.0 (0.0 - 2.0) | | 1.0 (0.0 - 2.0) | | 0.121 | |
| Time between previous surgery and index infection | | 52.5 (26.8 - 215.8) | | 48.0 (26.0 - 279.0) | | 52.0 (26.0 - 230.0) | | 0.867 | |
| Previous Surgery < 1 month | | 32 (29.6%) | | 10 (30.3%) | | 42 (29.8%) | | 0.941 | |
| Previous Surgery < 3 months | | 69 (63.9%) | | 20 (60.6%) | | 89 (63.1%) | | 0.732 | |
| Previous infection | | 89 (76.7%) | | 22 (61.1%) | | 111 (73.0%) | | 0.065 | |
| Previous infection due to *Candida* spp. | | 3 (3.3%) | | 3 (13.0%) | | 6 (5.3%) | | 0.095 | |
| **Microbiology analysis of previous infections** | |  | |  | |  | |  | |
| Mono-bacterial infection | | 42 (50.0%) | | 11 (55.0%) | | 53 (51.0%) | | 0.688 | |
| *Staphylococcus* sp. | | 60 (71.4%) | | 16 (80.0%) | | 76 (73.1%) | | 0.437 | |
| *Staphylococcus aureus* | | 28 (33.3%) | | 6 (30.0%) | | 34 (32.7%) | | 0.775 | |
| Coagulase-negative staphylococci | | 40 (47.6%) | | 10 (50.0%) | | 50 (48.1%) | | 0.848 | |
| *Streptococcus* sp | | 3 (2.7%) | | 3 (8.8%) | | 6 (4.1%) | | 0.141 | |
| *Enterococcus* sp. | | 12 (14.3%) | | 3 (15.0%) | | 15 (14.4%) | | >0.999 | |
| *Acinetobacter* sp. | | 3 (3.6%) | | 0 (0.0%) | | 3 (2.9%) | | >0.999 | |
| *Pseudomonas aeruginosa* | | 9 (10.7%) | | 4 (20.0%) | | 13 (12.5%) | | 0.269 | |
| Enterobacterales | | 31 (36.9%) | | 8 (40.0%) | | 39 (37.5%) | | 0.797 | |
| Corynebacteria | | 4 (3.6%) | | 1 (2.9%) | | 5 (3.4%) | | >0.999 | |
| Anaerobes | | 6 (5.4%) | | 2 (5.9%) | | 8 (5.5%) | | >0.999 | |
| Previous antibiotic therapy | | 85 (95.5%) | | 21 (95.5%) | | 106 (95.5%) | | >0.999 | |
| Number of lines of ATB | | 1.0 (1.0 - 2.0) | | 1.0 (1.0 - 2.5) | | 1.0 (1.0 - 2.0) | | 0.771 | |
| Previous ATB duration | | 47.5 (30.0 - 84.0) | | 39.5 (28.5 - 61.0) | | 45.0 (30.0 - 83.3) | | 0.275 | |
| **Clinical signs** | |  | |  | |  | |  | |
| Fever | | 21 (19.4%) | | 8 (22.2%) | | 29 (20.1%) | | 0.719 | |
| Inflammatory signs | | 62 (55.4%) | | 21 (60.0%) | | 83 (56.5%) | | 0.629 | |
| Purulent discharge | | 2 (2.4%) | | 4 (17.4%) | | 6 (5.7%) | | 0.019* | |
| Dehiscence | | 42 (37.2%) | | 11 (33.3%) | | 53 (36.3%) | | 0.687 | |
| Fistula | | 45 (39.8%) | | 12 (35.3%) | | 57 (38.8%) | | 0.635 | |
| Hematoma | | 17 (15.6%) | | 6 (18.8%) | | 23 (16.3%) | | 0.671 | |
| **Biological analysis** | |  | |  | |  | |  | |
| Leucocyte count (G/L) | | 7.6 (6.5 - 9.2) | | 8.0 (6.9 - 10.8) | | 7.7 (6.6 - 9.5) | | 0.281 | |
| Neutrophil count (G/L) | | 5.0 (3.7 - 6.3) | | 5.5 (3.9 - 6.8) | | 5.1 (3.8 - 6.4) | | 0.287 | |
| C-Reactive Protein level (mg/L) | | 33.5 (14.3 - 67.9) | | 47.0 (17.1 - 92.6) | | 35.0 (14.4 - 77.3) | | 0.254 | |
| ESR (mm/h) | | 66.0 (50.0 - 85.0) | | 35.0 (34.0 - 41.0) | | 62.5 (41.3 - 83.8) | | 0.066 | |
| Albumin level (g/L) | | 31.3 (23.8 - 38.1) | | 23.5 (20.0 - 31.0) | | 30.0 (22.0 - 35.0) | | 0.024* | |
| **Radiographic evidence of infection** | |  | |  | |  | |  | |
| X-ray | | 44 (42.7%) | | 12 (41.4%) | | 56 (42.4%) | | 0.897 | |
| CT scan | | 16 (29.6%) | | 5 (38.5%) | | 21 (31.3%) | | 0.526 | |
| Scintigraphy | | 2 (4.7%) | | 1 (8.3%) | | 3 (5.5%) | | 0.530 | |
| Loosening | | 20 (22.7%) | | 3 (11.5%) | | 23 (20.2%) | | 0.212 | |
| Abscess | | 14 (15.7%) | | 5 (20.0%) | | 19 (16.7%) | | 0.561 | |
| TTE/TOE | | 4 (4.9%) | | 5 (22.7%) | | 9 (8.7%) | | 0.019 | |
| Signs of endocarditis | | 0 (0.0%) | | 1 (5.0%) | | 1 (1.3%) | | 0.253 | |
| **Type of surgery** | |  | |  | |  | |  | |
| DAIR | | 42 (36.5%) | | 15 (42.9%) | | 57 (38.0%) | | 0.499 | |
| Exchange surgery | | 70 (60.9%) | | 17 (48.6%) | | 87 (58.0%) | | 0.197 | |
| One-stage exchange | | 32 (27.8%) | | 8 (22.9%) | | 40 (26.7%) | | 0.561 | |
| Two-stage exchange | | 38 (33.0%) | | 9 (25.7%) | | 47 (31.3%) | | 0.413 | |
| Other type of surgery | | 3 (2.6%) | | 3 (8.6%) | | 6 (4.0%) | | 0.140 | |
| **Microbiology analysis of index infection** | |  | |  | |  | |  | |
| Positive blood culture | | 12 (10.6%) | | 7 (20.0%) | | 19 (12.8%) | | 0.157 | |
| Pluri-microbial | | 52 (44.8%) | | 23 (63.9%) | | 75 (49.3%) | | 0.046* | |
| Only due to *Candida* spp. | | 59 (50.9%) | | 10 (27.8%) | | 69 (45.4%) | | 0.015 | |
| *C. albicans* | | 62 (53.4%) | | 18 (50.0%) | | 80 (52.6%) | | 0.717 | |
| *C. parapsilosis* | | 36 (31.0%) | | 10 (27.8%) | | 46 (30.3%) | | 0.710 | |
| Other *Candida* spp. | | 19 (16.4%) | | 9 (25.0%) | | 28 (18.4%) | | 0.244 | |
| **Co-infection with bacteria** | | 57 (49.1%) | | 26 (72.2%) | | 83 (54.6%) | | 0.015* | |
| *Staphylococcus* sp. | | 27 (25.5%) | | 19 (54.3%) | | 46 (32.6%) | | 0.002* | |
| *S. aureus* | | 7 (6.6%) | | 9 (25.7%) | | 16 (11.3%) | | 0.004* | |
| Coagulase-negative staphylococci | | 21 (19.8%) | | 13 (37.1%) | | 34 (24.1%) | | 0.038* | |
| *Enterococcus* sp | | 9 (8.5%) | | 3 (8.6%) | | 12 (8.5%) | | >0.999 | |
| *Streptococcus* sp. | | 0 (0.0%) | | 1 (2.9%) | | 1 (0.7%) | | 0.248 | |
| Enterobacterales | | 15 (14.2%) | | 7 (20.0%) | | 22 (15.6%) | | 0.408 | |
| *E. coli* | | 3 (2.8%) | | 4 (11.4%) | | 7 (5.0%) | | 0.064 | |
| *K. pneumoniae* | | 3 (2.8%) | | 1 (2.9%) | | 4 (2.8%) | | >0.999 | |
| *Enterobacter* sp. | | 7 (6.6%) | | 2 (5.7%) | | 9 (6.4%) | | >0.999 | |
| *Pseudomonas aeruginosa* | | 3 (2.8%) | | 2 (5.7%) | | 5 (3.5%) | | 0.598 | |
| Corynebacteria | | 4 (3.8%) | | 2 (5.7%) | | 6 (4.3%) | | 0.638 | |
| Anaerobes | | 2 (1.9%) | | 1 (2.9%) | | 3 (2.1%) | | >0.999 | |
| **Antifungal susceptibility testing** | |  | |  | |  | |  | |
| Resistance to Fluconazole | | 11 (10.4%) | | 1 (3.6%) | | 12 (9.0%) | | 0.459 | |
| Resistance to Voriconazole | | 4 (4.2%) | | 0 (0.0%) | | 4 (3.3%) | | 0.579 | |
| Resistance to Posaconazole | | 3 (9.4%) | | 0 (0.0%) | | 3 (7.7%) | | >0.999 | |
| Resistance to Echinocandins | | 11 (11.8%) | | 4 (16.7%) | | 15 (12.8%) | | 0.506 | |
| Resistance to 5-fluorocytosine | | 2 (3.7%) | | 0 (0.0%) | | 2 (3.2%) | | >0.999 | |
| **Antifungal treatments** | |  | |  | |  | |  | |
| Number of lines | | 2.0 (1.0 - 2.0) | | 1.0 (1.0 - 1.0) | | 1.0 (1.0 - 2.0) | | <0.001* | |
| Antibiofilm > 1 week | | 44 (37.9%) | | 9 (25.0%) | | 53 (34.9%) | | 0.155 | |
| Azoles | | 102 (87.9%) | | 25 (69.4%) | | 127 (83.6%) | | 0.009* | |
| Echinocandins | | 44 (37.9%) | | 10 (27.8%) | | 54 (35.5%) | | 0.266 | |
| Azoles & Echinocandins | | 10 (8.6%) | | 0 (0.0%) | | 10 (6.6%) | | 0.118 | |
| Amphotericin B | | 9 (7.8%) | | 0 (0.0%) | | 9 (5.9%) | | 0.116 | |
| Echinocandins & 5-flucytosine | | 5 (4.3%) | | 1 (2.8%) | | 6 (3.9%) | | >0.999 | |
| Azoles & 5-flucytosine | | 3 (2.6%) | | 0 (0.0%) | | 3 (2.0%) | | >0.999 | |
| Antifungal treatment duration | | 169.5 (104.3 - 201.5) | | 29.5 (14.0 - 37.3) | | 120.0 (85.0 - 187.8) | | <0.001* | |
| Antifungal-impregnated cement spacer | | 6 (5.2%) | | 0 (0.0%) | | 6 (3.9%) | | 0.336 | |
| **Percentage of total treatment time** | |  | |  | |  | |  | |
| Azoles | | 100.0 (86.2 - 100.0) | | 100.0 (100.0 - 100.0) | | 100.0 (87.1 - 100.0) | | <0.001* | |
| Echinocandins | | 13.5 (7.2 - 53.2) | | 100.0 (100.0 - 100.0) | | 19.6 (8.0 - 100.0) | | <0.001* | |
| Azoles & Echinocandins | | 11.5 (6.8 - 13.7) | | NA (NA - NA) | | 11.5 (6.8 - 13.7) | |  | |
| Amphotericin B | | 13.2 (7.0 - 14.5) | | NA (NA - NA) | | 13.2 (7.0 - 14.5) | |  | |
| Echinocandins & 5-flucytosine | | 20.5 (18.1 - 51.3) | | 100.0 (100.0 - 100.0) | | 35.9 (18.7 - 87.8) | | 0.373 | |
| Azoles & 5-flucytosine | | 61.1 (41.3 - 67.6) | | NA (NA - NA) | | 61.1 (41.3 - 67.6) | |  | |
| Concomitant antibiotic therapy | | 87 (75.0%) | | 33 (91.7%) | | 120 (78.9%) | | 0.032* | |
| Concomitant antibiotic treatment duration | | 68.5 (42.0 - 84.0) | | 42.0 (25.0 - 45.0) | | 45.0 (40.0 - 84.0) | | 0.003* | |
| **Outcome** | |  | |  | |  | |  | |
| Cure | | 69 (59.5%) | | 18 (50.0%) | | 87 (57.2%) | | 0.315 | |
| Recurrence other germs | | 28 (24.1%) | | 5 (13.9%) | | 33 (21.7%) | | 0.193 | |
| Failure | | 47 (40.5%) | | 18 (50.0%) | | 65 (42.8%) | | 0.315 | |
| Suppressive treatment | | 11 (9.5%) | | 0 (0.0%) | | 11 (7.2%) | | 0.067 | |
| Recurrence | | 28 (24.1%) | | 5 (13.9%) | | 33 (21.7%) | | 0.193 | |
| Recurrence to *Candida* spp. | | 11 (9.5%) | | 3 (8.3%) | | 14 (9.2%) | | >0.999 | |
| Recurrence not documented | | 9 (7.8%) | | 1 (2.8%) | | 10 (6.6%) | | 0.453 | |
| Recurrence to *Candida* spp. and other bacteria | | 8 (6.9%) | | 1 (2.8%) | | 9 (5.9%) | | 0.687 | |
| Death due to infectious cause | | 6 (5.2%) | | 9 (25.0%) | | 15 (9.9%) | | 0.002* | |
| Death due to other cause | | 2 (1.7%) | | 4 (11.1%) | | 6 (3.9%) | | 0.028* | |
| Follow-up period | | 675.0 (377.0 - 1,282.0) | | 359.0 (115.5 - 732.5) | | 578.0 (315.5 - 1,197.0) | | 0.004* | |

^1^ Median (IQR1 - IQR3); n (%)

^2^ Pearson's Chi-squared test; Fisher's exact test; Wilcoxon rank sum exact test

*Statistically significant

Table S11. Patients' characteristics according to antifungal duration (6-12 weeks *versus* > 12 weeks)

|  | 6 - 12 Weeks  N = 44^1^ | > 12 Weeks  N = 116^1^ | Overall  N = 160^1^ | P-value^2^ |
| --- | --- | --- | --- | --- |
| Age (year) | 75.0 (66.8 - 82.0) | 71.0 (61.0 - 77.0) | 71.0 (61.8 - 78.3) | 0.043* |
| Age > 70 yo | 26 (59.1%) | 62 (53.4%) | 88 (55.0%) | 0.522 |
| Body Mass Index | 30.8 (26.5 - 34.7) | 29.4 (24.5 - 34.4) | 29.9 (24.9 - 34.4) | 0.389 |
| Charlson score | 4.0 (2.0 - 5.0) | 3.0 (2.0 - 5.0) | 3.0 (2.0 - 5.0) | 0.210 |
| Male patients | 20 (45.5%) | 63 (54.3%) | 83 (51.9%) | 0.317 |
| Immunosuppression | 2 (4.5%) | 16 (13.8%) | 18 (11.3%) | 0.159 |
| Immunosuppressive treatments | 3 (8.1%) | 12 (10.6%) | 15 (10.0%) | >0.999 |
| Diabetes | 13 (29.5%) | 33 (28.4%) | 46 (28.8%) | 0.891 |
| **Localization of prosthesis** |  |  |  |  |
| Hip | 26 (59.1%) | 58 (50.0%) | 84 (52.5%) | 0.304 |
| Knee | 16 (36.4%) | 53 (45.7%) | 69 (43.1%) | 0.288 |
| Other | 2 (4.5%) | 5 (4.3%) | 7 (4.4%) | >0.999 |
| Number of previous surgeries | 3.0 (2.0 - 5.0) | 3.0 (2.0 - 5.0) | 3.0 (2.0 - 5.0) | 0.695 |
| Number of previous surgeries due to infection | 1.0 (1.0 - 2.0) | 1.0 (0.0 - 2.0) | 1.0 (1.0 - 2.0) | 0.579 |
| Time between previous surgery and index infection | 105.0 (21.0 - 598.5) | 52.5 (26.8 - 215.8) | 55.0 (24.0 - 373.5) | 0.746 |
| Previous Surgery < 1 month | 17 (39.5%) | 32 (29.6%) | 49 (32.5%) | 0.241 |
| Previous Surgery < 3 months | 21 (48.8%) | 69 (63.9%) | 90 (59.6%) | 0.089 |
| Previous infection | 37 (84.1%) | 89 (76.7%) | 126 (78.8%) | 0.309 |
| Previous infection due to *Candida* spp. | 3 (7.9%) | 3 (3.3%) | 6 (4.7%) | 0.359 |
| **Microbiology analysis of previous infections** |  |  |  |  |
| Mono-bacterial infection | 18 (54.5%) | 42 (50.0%) | 60 (51.3%) | 0.658 |
| *Staphylococcus* sp. | 16 (48.5%) | 60 (71.4%) | 76 (65.0%) | 0.019* |
| *Staphylococcus aureus* | 5 (15.2%) | 28 (33.3%) | 33 (28.2%) | 0.049* |
| Coagulase-negative staphylococci | 13 (39.4%) | 40 (47.6%) | 53 (45.3%) | 0.421 |
| *Streptococcus* sp | 4 (10.0%) | 3 (2.7%) | 7 (4.6%) | 0.080 |
| *Enterococcus* sp. | 5 (15.2%) | 12 (14.3%) | 17 (14.5%) | >0.999 |
| *Acinetobacter* sp. | 0 (0.0%) | 3 (3.6%) | 3 (2.6%) | 0.558 |
| *Pseudomonas aeruginosa* | 7 (21.2%) | 9 (10.7%) | 16 (13.7%) | 0.147 |
| Enterobacterales | 10 (30.3%) | 31 (36.9%) | 41 (35.0%) | 0.501 |
| Corynebacteria | 1 (2.5%) | 4 (3.6%) | 5 (3.3%) | >0.999 |
| Anaerobes | 3 (7.5%) | 6 (5.4%) | 9 (6.0%) | 0.700 |
| Previous antibiotic therapy | 35 (94.6%) | 85 (95.5%) | 120 (95.2%) | >0.999 |
| Number of lines of ATB | 1.0 (1.0 - 2.0) | 1.0 (1.0 - 2.0) | 1.0 (1.0 - 2.0) | 0.818 |
| Previous ATB duration | 49.0 (43.0 - 81.5) | 47.5 (30.0 - 84.0) | 48.0 (31.0 - 84.0) | 0.459 |
| **Clinical signs** |  |  |  |  |
| Fever | 4 (9.3%) | 21 (19.4%) | 25 (16.6%) | 0.130 |
| Inflammatory signs | 15 (35.7%) | 62 (55.4%) | 77 (50.0%) | 0.030* |
| Purulent discharge | 0 (0.0%) | 2 (2.4%) | 2 (1.8%) | >0.999 |
| Dehiscence | 14 (32.6%) | 42 (37.2%) | 56 (35.9%) | 0.592 |
| Fistula | 16 (38.1%) | 45 (39.8%) | 61 (39.4%) | 0.845 |
| Hematoma | 8 (19.0%) | 17 (15.6%) | 25 (16.6%) | 0.609 |
| **Biological analysis** |  |  |  |  |
| Leucocyte count (G/L) | 7.2 (5.8 - 8.6) | 7.6 (6.5 - 9.2) | 7.5 (6.2 - 8.9) | 0.182 |
| Neutrophil count (G/L) | 4.5 (3.8 - 6.0) | 5.0 (3.7 - 6.3) | 4.8 (3.7 - 6.2) | 0.700 |
| C-Reactive Protein level (mg/L) | 25.2 (8.1 - 54.4) | 33.5 (14.3 - 67.9) | 29.0 (12.2 - 66.3) | 0.177 |
| ESR (mm/h) | 55.5 (17.0 - 85.0) | 66.0 (50.0 - 85.0) | 65.0 (43.5 - 87.5) | 0.353 |
| Albumin level (g/L) | 33.0 (28.5 - 36.0) | 31.3 (23.8 - 38.1) | 32.0 (25.0 - 37.0) | 0.428 |
| **Radiographic evidence of infection** |  |  |  |  |
| X-ray | 19 (47.5%) | 44 (42.7%) | 63 (44.1%) | 0.605 |
| CT scan | 2 (16.7%) | 16 (29.6%) | 18 (27.3%) | 0.487 |
| Scintigraphy | 2 (20.0%) | 2 (4.7%) | 4 (7.5%) | 0.157 |
| Loosening | 5 (17.9%) | 20 (22.7%) | 25 (21.6%) | 0.585 |
| Abscess | 2 (7.7%) | 14 (15.7%) | 16 (13.9%) | 0.519 |
| TTE/TOE | 3 (10.3%) | 4 (4.9%) | 7 (6.3%) | 0.375 |
| **Type of surgery** |  |  |  |  |
| DAIR | 20 (45.5%) | 42 (36.5%) | 62 (39.0%) | 0.302 |
| Exchange surgery | 22 (50.0%) | 70 (60.9%) | 92 (57.9%) | 0.214 |
| One-stage exchange | 8 (18.2%) | 32 (27.8%) | 40 (25.2%) | 0.210 |
| Two-stage exchange | 14 (31.8%) | 38 (33.0%) | 52 (32.7%) | 0.883 |
| Other type of surgery | 2 (4.5%) | 3 (2.6%) | 5 (3.1%) | 0.617 |
| **Microbiology analysis of index infection** |  |  |  |  |
| Positive blood culture | 3 (7.0%) | 12 (10.6%) | 15 (9.6%) | 0.762 |
| Pluri-microbial | 22 (50.0%) | 52 (44.8%) | 74 (46.3%) | 0.558 |
| Only due to *Candida* spp. | 21 (47.7%) | 59 (50.9%) | 80 (50.0%) | 0.723 |
| *C. albicans* | 28 (63.6%) | 62 (53.4%) | 90 (56.3%) | 0.246 |
| *C. parapsilosis* | 12 (27.3%) | 36 (31.0%) | 48 (30.0%) | 0.643 |
| Other *Candida* spp. | 7 (15.9%) | 19 (16.4%) | 26 (16.3%) | 0.943 |
| **Co-infection with bacteria** | 23 (52.3%) | 57 (49.1%) | 80 (50.0%) | 0.723 |
| *Staphylococcus* sp. | 9 (22.5%) | 27 (25.5%) | 36 (24.7%) | 0.710 |
| *S. aureus* | 2 (5.0%) | 7 (6.6%) | 9 (6.2%) | >0.999 |
| Coagulase-negative staphylococci | 7 (17.5%) | 21 (19.8%) | 28 (19.2%) | 0.752 |
| *Enterococcus* sp | 5 (12.5%) | 9 (8.5%) | 14 (9.6%) | 0.531 |
| *Streptococcus* sp. | 1 (2.5%) | 0 (0.0%) | 1 (0.7%) | 0.274 |
| Enterobacterales | 7 (17.5%) | 15 (14.2%) | 22 (15.1%) | 0.614 |
| *E. coli* | 0 (0.0%) | 3 (2.8%) | 3 (2.1%) | 0.562 |
| *K. pneumoniae* | 4 (10.0%) | 3 (2.8%) | 7 (4.8%) | 0.090 |
| *Enterobacter* sp. | 1 (2.5%) | 7 (6.6%) | 8 (5.5%) | 0.446 |
| *Pseudomonas aeruginosa* | 2 (5.0%) | 3 (2.8%) | 5 (3.4%) | 0.615 |
| Corynebacteria | 1 (2.5%) | 4 (3.8%) | 5 (3.4%) | >0.999 |
| Anaerobes | 3 (7.5%) | 2 (1.9%) | 5 (3.4%) | 0.127 |
| **Antifungal susceptibility testing** |  |  |  |  |
| Resistance to Fluconazole | 3 (7.7%) | 11 (10.4%) | 14 (9.7%) | 0.760 |
| Resistance to Voriconazole | 2 (6.1%) | 4 (4.2%) | 6 (4.7%) | 0.647 |
| Resistance to Posaconazole | 1 (7.1%) | 3 (9.4%) | 4 (8.7%) | >0.999 |
| Resistance to Echinocandins | 2 (6.5%) | 11 (11.8%) | 13 (10.5%) | 0.515 |
| Resistance to 5-fluorocytosine | 3 (16.7%) | 2 (3.7%) | 5 (6.9%) | 0.096 |
| **Antifungal treatments** |  |  |  |  |
| Number of lines | 1.0 (1.0 - 2.0) | 2.0 (1.0 - 2.0) | 1.0 (1.0 - 2.0) | 0.113 |
| Antibiofilm > 1 week | 13 (29.5%) | 44 (37.9%) | 57 (35.6%) | 0.323 |
| Azoles | 36 (81.8%) | 102 (87.9%) | 138 (86.3%) | 0.316 |
| Echinocandins | 13 (29.5%) | 44 (37.9%) | 57 (35.6%) | 0.323 |
| Azoles & Echinocandins | 5 (11.4%) | 10 (8.6%) | 15 (9.4%) | 0.558 |
| Amphotericin B | 6 (13.6%) | 9 (7.8%) | 15 (9.4%) | 0.361 |
| Echinocandins & 5-flucytosine | 0 (0.0%) | 5 (4.3%) | 5 (3.1%) | 0.324 |
| Azoles & 5-flucytosine | 3 (6.8%) | 3 (2.6%) | 6 (3.8%) | 0.347 |
| Antifungal treatment duration | 60.5 (53.0 - 65.5) | 169.5 (104.3 - 201.5) | 116.0 (79.8 - 186.0) | <0.001* |
| Antifungal-impregnated cement spacer | 4 (9.1%) | 6 (5.2%) | 10 (6.3%) | 0.464 |
| **Percentage of total treatment time** |  |  |  |  |
| Azoles | 100.0 (75.8 - 100.0) | 100.0 (86.2 - 100.0) | 100.0 (82.6 - 100.0) | 0.748 |
| Echinocandins | 48.8 (19.0 - 100.0) | 13.5 (7.2 - 53.2) | 19.0 (8.1 - 78.3) | 0.009* |
| Azoles & Echinocandins | 26.6 (14.0 - 26.9) | 11.5 (6.8 - 13.7) | 13.4 (9.6 - 26.7) | 0.075 |
| Amphotericin B | 32.4 (27.5 - 48.1) | 13.2 (7.0 - 14.5) | 14.5 (10.5 - 28.1) | 0.002* |
| Echinocandins & 5-flucytosine | NA (NA - NA) | 20.5 (18.1 - 51.3) | 20.5 (18.1 - 51.3) |  |
| Azoles & 5-flucytosine | 56.4 (37.7 - 78.2) | 61.1 (41.3 - 67.6) | 58.7 (30.3 - 70.9) | >0.999 |
| Concomitant antibiotic therapy | 32 (72.7%) | 87 (75.0%) | 119 (74.4%) | 0.769 |
| Concomitant antibiotic treatment duration | 56.0 (42.0 - 84.0) | 68.5 (42.0 - 84.0) | 60.5 (42.0 - 84.0) | 0.592 |
| **Outcome** |  |  |  |  |
| Cure | 29 (65.9%) | 69 (59.5%) | 98 (61.3%) | 0.456 |
| Recurrence other germs | 12 (27.3%) | 28 (24.1%) | 40 (25.0%) | 0.683 |
| Failure | 15 (34.1%) | 47 (40.5%) | 62 (38.8%) | 0.456 |
| Suppressive treatment | 0 (0.0%) | 11 (9.5%) | 11 (6.9%) | 0.036* |
| Recurrence | 8 (18.2%) | 28 (24.1%) | 36 (22.5%) | 0.420 |
| Recurrence to *Candida* spp. | 5 (11.4%) | 11 (9.5%) | 16 (10.0%) | 0.770 |
| Recurrence not documented | 1 (2.3%) | 9 (7.8%) | 10 (6.3%) | 0.287 |
| Recurrence to *Candida* spp. and other bacteria | 2 (4.5%) | 8 (6.9%) | 10 (6.3%) | 0.728 |
| Death due to infectious cause | 5 (11.4%) | 6 (5.2%) | 11 (6.9%) | 0.176 |
| Death due to other cause | 2 (4.5%) | 2 (1.7%) | 4 (2.5%) | 0.304 |
| Follow-up period | 746.0 (252.0 - 1,504.0) | 675.0 (377.0 - 1,282.0) | 675.0 (362.8 - 1,308.3) | 0.696 |

^1^ Median (IQR1 - IQR3); n (%)

^2^ Pearson's Chi-squared test; Fisher's exact test; Wilcoxon rank sum exact test

*Statistically significant

## Analysis on antifungal sensibility according to *Candida* species

Table S12. Antifungal sensibility analysis according to *Candida* species

| **Resistance to antifungal tested** | **Overall**  N = 234^1^ | ***C. albicans* versus other *Candida* spp.** | | | ***C. parapsilosis* versus other *Candida* spp.** | | | |
| --- | --- | --- | --- | --- | --- | --- | --- | --- |
|  |  | ***C. albicans***  N = 128^1^ | **Other *Candida* spp.**  N = 106^1^ | **p-value**^2^ | ***C. parapsilosis***  N = 74^1^ | **Other *Candida* spp.**  N = 160^1^ | **p-value**^2^ |  |
| Fluconazole | 21/234 (9.0%) | 8/128 (6.3%) | 13/106 (12%) | 0.109 | 3/74 (4.1%) | 18/160 (11%) | 0.073 |  |
| Voriconazole | 11/206 (5.3%) | 6/111 (5.4%) | 5/95 (5.3%) | 0.964 | 0/68 (0%) | 11/138 (8.0%) | 0.017 |  |
| Posaconazole | 5/60 (8.3%) | 2/35 (5.7%) | 3/25 (12%) | 0.640 | 1/18 (5.6%) | 4/42 (9.5%) | >0.999 |  |
| Amphotericin B | 4/218 (1.8%) | 1/117 (0.9%) | 3/101 (3.0%) | 0.339 | 3/71 (4.2%) | 1/147 (0.7%) | 0.102 |  |
| Echinocandins | 29/201 (14%) | 1/112 (0.9%) | 28/89 (31%) | <0.001 | 28/63 (44%) | 1/138 (0.7%) | <0.001 |  |
| 5-fluorocytosine | 5/97 (5.2%) | 2/57 (3.5%) | 3/40 (7.5%) | 0.401 | 0/23 (0%) | 5/74 (6.8%) | 0.335 |  |
| Ketoconazole | 0/1 (0%) | 0/0 (NA%) | 0/1 (0%) |  | 0/1 (0%) | 0/0 (NA%) |  |  |
| Micafungine | 0/3 (0%) | 0/2 (0%) | 0/1 (0%) |  | 0/1 (0%) | 0/2 (0%) |  |  |
| Itraconazole | 1/10 (10%) | 1/6 (17%) | 0/4 (0%) | >0.999 | 0/2 (0%) | 1/8 (13%) | >0.999 |  |

^1^ n (%)

^2^ Pearson’s Chi-squared test; Fisher’s exact test
